# Brownmillerite Calcium Ferrite, a Promising Perovskite‐Related Material in the Degradation of a Tight Dye under Ambient Conditions

**DOI:** 10.1002/open.202300169

**Published:** 2023-12-05

**Authors:** Zahra Noori, Azim Malekzadeh, Jordi Poater

**Affiliations:** ^1^ School of Chemistry Damghan University Damghan 367126/41167 Iran; ^2^ Departament de Química Inorgànica i Orgànica & IQTCUB Universitat de Barcelona Martí i Franquès 1–11 08028 Barcelona Spain; ^3^ ICREA Passeig Lluís Companys 23 08010 Barcelona Spain

**Keywords:** Alizarin Red S, Brownmillerite, Calcium ferrite, Dye degradation, Tight dye

## Abstract

Evaluation of effective and low‐cost materials as catalysts to combat the threat of pollution is a significant and growing trend. With this aim, we have synthesized calcium ferrite brownmillerite by wet preparation approach as a catalyst for pollution. The structural analysis is established by the X‐ray diffraction of Ca_2_Fe_2_O_5_, whereas the tetrahedral and octahedral sites band stretching for ferrite specimen has been deduced using FTIR. The bandgap energy has been estimated by the Tauc relation (2.17 eV). Ca_2_Fe_2_O_5_ brownmillerite exhibits a BET surface area of 10 m^2^/g and a BJH pore volume of 0.121 cm^3^/g with the average particle size of 70 nm. Importantly, the alizarin Red S dye degradation has been studied using the prepared ferrite catalyst, under dark ambient conditions and without the presence of any acidic or basic additives. Degradation is also supported by both FTIR and TOC analysis. Surface properties of brownmillerite Ca_2_Fe_2_O_5_ have been characterized using electronic spectroscopy and CO_2_ temperature programmed desorption (TPD) analysis and revealed that the basic surface of brownmillerite Ca_2_Fe_2_O_5_ offers active sites that are suitable for degradation processes. All results show that the preparation of brownmillerite Ca_2_Fe_2_O_5_ via the Pechini method is suitable to produce fine surfaces and pores with nanosized particles.

## Graphical abstract

Synthesized and characterized brownmillerite type Ca_2_Fe_2_O_5_ nanoparticles degrade Alizarin Red S (ARS) quinone dye in the darkness, making it a promising material for the degradation of not only ARS but of other persistent dyes, too.

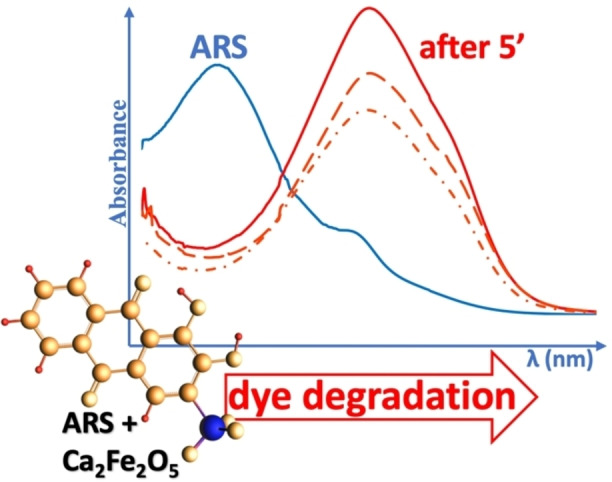

## Introduction

Industrial chemicals wastewater containing hazardous dyes cause problems for human life, animal life, as well as aquatic life.^[1]^ Compared to other chemical approaches, the degradation technique, whether catalytic or photocatalytic, has been proven to be significantly superior among the methods utilized for purifying contaminated water.^[2]^ While various binary catalysts based on transition metals exhibit relatively high activity, their extensive use is not environmentally desirable due to their toxicity and high consumption. As a result, it is crucial to move to cheaper and safer catalysts that employ more sustainable metals.

Perovskite oxides are common structures in inorganic chemistry that are often considered for use as catalysts and supports.^[3]^ A perovskite structure that conforms to the general formula ABO_3_ typically features a cubic crystal structure consisting of a framework of BO_6_ octahedra, which share corners in three dimensions.^[4]^ The properties exhibited by perovskite oxides are mainly attributed to the structure of the BO_6_ octahedral network and the state of the cations located at the B‐site.^[5]^

Brownmillerite materials belong to the family of perovskite‐related compounds and possess the chemical formula A_2_B_2_O_5_.^[6]^ The mineral with the chemical formula Ca_2_(Al,Fe)_2_O_5_ is a scarcely occurring oxide and was named after Lorrin Thomas Brownmiller (1902–1990), who served as the chief chemist for the Alpha Portland Cement Company in Easton, Pennsylvania. The perovskite structure (ABO_2.5_) lacking oxygen atoms is characterized by a three‐dimensional framework composed of BO_6_ octahedra sharing corners, interspersed with slabs containing rows of BO_4_ tetrahedra sharing corners. The oxygen deficiency during the structure formation process leads to the formation of these tetrahedral slabs. In other words, the brownmillerite oxides can be characterized as having a perovskite structure^[7]^ with a regularly arranged pattern of one‐sixth of the oxygen atoms being absent.^[8]^ This type of imperfection renders the brownmillerite structure resilient and unresponsive to external factors, particularly exposure to light. The oxygen non‐stoichiometry of brownmillerite is also the foundation for its application as a catalyst in the partial oxidation of hydrocarbons.^[9]^

Ferrite nanomaterials have become widely used in the treatment of wastewater, and the significance of brownmillerite‐type ferrites as catalysts or photocatalysts in environmental applications has been demonstrated. In the current research, we have prepared calcium ferrite (CaFeO_2.5_), that exemplifies a stoichiometric phase of brownmillerite,^[10]^ through a bottom‐up approach using a complexation route and examined its effectiveness in breaking down alizarin Red S (ARS) dye. According to the findings, under regular conditions without the need for additional substances or light exposure, nanoparticles with a brownmillerite‐type ferrite structure exhibit suitable catalytic activity. The structural characteristics of this ferrite type have also been examined in the present analysis. Further insight into the intriguing reaction of ARS with calcium ferrite brownmillerite has been obtained through a quantum chemical analysis.

## Materials and Methods

### Catalyst preparation

The first step was the obtention of brownmillerite type Ca_2_Fe_2_O_5_ nanoparticles. For such, analytical reagents Ca(NO_3_)_2_ ⋅ 4H_2_O, Fe(NO_3_)_2_ ⋅ 9H_2_O, and citric acid (C_6_H_6_O_7_ ⋅ H_2_O) from Merk were added to 20 mL of distilled water in a molar ratio of 1 : 1 : 5. Keeping the solution at a constant 50 °C, it was stirred continuously for 4 hours. After stirring, the solution was dried in a 60 °C oven overnight. Separately, the prepared gel was placed in an 80 °C oven overnight. The sponge material underwent a complete grinding process, followed by drying at 160 °C overnight and subsequently being powdered. As the drying process took place, the reactants underwent both decomposition and combustion reactions. Following the auto‐combustion process, a solid material was obtained and then pulverized into a fine powder using an agate pestle and mortar. Calcination of the fine powder was carried out at 750 °C for 6 hours in a muffle furnace to obtain brownmillerite type Ca_2_Fe_2_O_5_ nanoparticles, referred to as BCFO hereafter. This compound was characterized with the following techniques described below.

### Catalyst characterization

To verify the sample‘s purity, an Oxford instrument was utilized to conduct an Energy Dispersive Spectroscopy (EDS) analysis. The Field Emission Scanning Electron Microscopy (FESEM) was employed to investigate the surface morphology of the synthesized nanoparticles (JSMIT300 SEM, JOEL Japan). The XRD data of the samples are recorded using the Cu Kα (λ=1.5418 Å) radiation for line scanning 10°–80° (2θ range) at a 1°/min rate using an X‐ray diffractometer (Bruker AXS diffractometer D8 ADVANCE with Cu Kα radiation). Fourier Transform Infrared (FTIR) spectroscopy was performed by PERKIN‐ELMER FT‐IR spectrometer over a wavenumber range of 400–2000 cm^−1^. The amount of total organic carbon (TOC) was analyzed by the organic matter combustion method (Multi N/C 3100 Analytical Jena). The ultraviolet visible diffused reflectance spectroscopy was also studied. The CO_2_‐TPD of freshly calcined catalysts was characterized by the temperature‐programmed desorption technique using a conventional TPD apparatus, Chemisorption Analyzer, NanoSORD (made by Sensiran Co., Iran). A gas stream of 10 % CO_2_ in helium with the flow rate of 10 mL/min through a quartz U tube containing 40 mg of catalyst was used for TPD analysis. Due to its small cross‐sectional area of 0.195 nm^2^, CO_2_ molecules are able to adsorb onto the basic sites of the catalyst. The CO_2_ TPD method can differentiate sites based on their sorption strength. The TPD curve shape of CO_2_ and the number of basic sites identified by the TPD test are heavily dependent on the experimental conditions. The CO_2_ TPD test was conducted directly on the fresh catalyst without any prior activation. The sample was degassed at 300 °C for 60 minutes before being exposed to a 10 % CO_2_/He mixture and adsorbed at 50 °C for 30 minutes. The TPD test was conducted with a helium flow rate of 10 sccm and a ramp rate of 10 °C/min, starting from 45 °C. After the adsorption process, the sample was flushed with pure helium for 1 h to remove the physically bound CO_2_ and stabilize the TCD signal before desorption was carried out. The surface area and pore volume of the sample were analyzed by BELSORP MINI II surface area analyzer (SAA) from the BEL company with N_2_ sorption isotherm technique. To eliminate physically adsorbed water and other impurities, the samples were degassed at 200 °C for 2 h prior to measurement. The adsorption of nitrogen gas was carried out on a BCFO sample at a bath temperature of 77 K, which is equivalent to the temperature of liquid nitrogen. The Brunauer‐Emmett‐Teller (BET) method was used to measure the specific surface area of the sample,^[11]^ while the pore size distribution was measured by Barrett‐Joyner‐Halenda (BJH) method.^[12]^ The bandgap of the BCFO sample was calculated using the Tauc plot. X‐ray photoemission spectroscopy (XPS) was performed at the Center for Research in Multiscale Science and Engineering of the UPC university in Barcelona. It was carried out using an X‐ray photoelectron spectroscopy chamber PHOIBOS 150 EP hemispherical energy analyzer with MCD‐9 detector, XR‐50 X‐ray source with a twin anode (Al, Mg), high pressure and high temperature chamber for gas treatments of the samples, sputtering system for cleaning and depth profiling, analysis Specs Lab Prodigy data acquisition and experiment control software package.

ARS, which stands for Alizarin Red S, is categorized as a tight dye due to its exceptional resistance to degradation. However, the catalytic performance of BCFO was examined in the degradation of ARS dye under ambient conditions without the use of any additives. Several equations can be used to describe the equilibrium adsorption of an adsorption process. In this study, the Langmuir, Freundlich, and Temkin isotherm equations were used to evaluate the removal of the dye from aqueous solution. The adsorption kinetics of ARS on BCFO were modeled using two common models: the pseudo‐first‐order and pseudo‐second‐order models.

**Figure 1:**
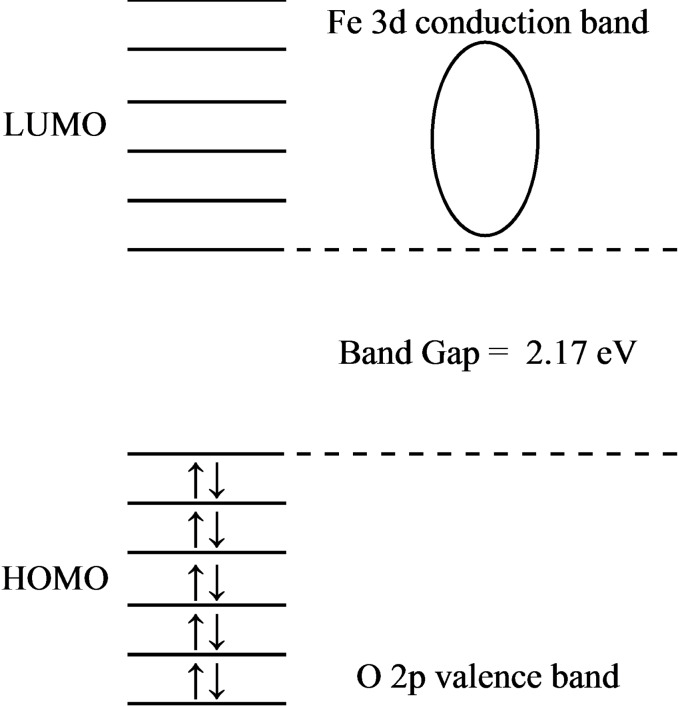
Schematic representation of how the band gap is defined in the MO form for BCFO.

***Bandgap calculation using Tauc plot***. The Tauc equation, referred to as the Tauc model or Tauc plot, mathematically expresses the correlation between the absorption coefficient of a substance and the energy level of the incoming photons. This equation finds frequent application in the realm of optical spectroscopy for studying the optical characteristics of materials such as semiconductors and insulators. The Tauc equation is given by Eq. 1:

(1) $${\left(Ah\upsilon \right)}^{\frac{1}{n}}=B\left(h\upsilon -Eg\right)$$

where A is the absorption coefficient, hν is the energy of the incident photons, B is a constant related to the density of states of the material and the strength of the electronic transition, Eg is the optical bandgap energy of the material, and n is the exponent that depends on the nature of the electronic transition responsible for the absorption (typically, n=1/2 for direct band gap materials and n=2 for indirect band gap materials). Direct band gap materials are a type of semiconductor materials that have a band gap energy that allows them to efficiently emit or absorb light. In these materials, the minimum energy required for an electron to move from the valence band to the conduction band (i. e., the band gap energy) is the same as the energy of a photon of light. Indirect bandgap materials are a type of semiconductor material in which the minimum energy required for an electron to move from the valence band to the conduction band is different from the energy of a photon of light. In these materials, the lowest energy state of the conduction band is not aligned with the highest energy state of the valence band, meaning that there is no direct transition between the two bands. As a result, indirect bandgap materials are less efficient in emitting or absorbing light than direct bandgap materials. When an electron in the conduction band recombines with a hole in the valence band, the energy is typically dissipated as heat rather than being emitted as a photon of light. Figure 1 represents how the band gap of the BCFO is defined in the MO form.

***Isothermal modeling of equilibrium data***. Adsorption isotherms are used in many different fields to characterize the adsorption behavior of the material. Dye distribution between the aqueous medium and adsorbent is a way to assess the equilibrium position in the adsorption process. Different isotherms quite agree with the experiment over restricted ranges of concentration, but they remain largely empirical. All the isotherms are useful in describing the adsorption of solutes onto surfaces in a solution, but they are based on different assumptions and models. Which model to use depends on the specific application and the characteristics of the adsorption process being studied. Here, Langmuir, Temkin, and Freundlich isotherms were used to describe the adsorption of ARS solute onto surface of brownmillerite CaFeO_2.5_ in solution.

The Langmuir isotherm assumes that the adsorption of solute onto the surface is a monolayer process, where the adsorbate molecules occupy a fixed number of sites on the surface, and the adsorption is proportional to the concentration of the solute in the solution (Eq. 2.

(2) $$\frac{C_{e}}{q_{e}}=\frac{1}{q_{max}}C_{e}+\frac{1}{q_{max}K_{L}}$$

where: C_e_=the concentration of solute in the solution (mg/L); q_e_=the amount of solute adsorbed per unit mass of adsorbent (mg/g); q_max_=the maximum adsorption capacity of the adsorbent (mg/g); and K_L_=the Langmuir constant related to the affinity of the solute for the adsorbent (L/mg).

Langmuir separation factor “R_L_”, which is related to the affinity of binding sites, and thermodynamical parameter ΔG° were calculated using Eqs. 3 and 4, respectively.

(3) $$R_{L}=\frac{1}{1+K_{L}{\cdot q}_{max}}$$

(4)

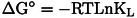




The R_L_ is a dimensionless equilibrium parameter and its value determines the type of adsorption isotherm that is observed. The 0<R_L_<1 is related to a favorable adsorption. The R_L_=1 and R_L_>1 are related to a linear or unfavorable adsorption, respectively.

The Freundlich isotherm can be used to model a wide range of adsorption systems, including the removal of organic pollutants, heavy metal ions or pharmaceuticals from aqueous solutions or wastewaters. Its basic assumption is that non‐uniform distribution of adsorbate on heterogeneous surface. The Freundlich isotherm is more appropriate for complex, multilayer adsorption processes with many adsorption sites. The equilibrium data applied to the Freundlich model is given in Eq. 5.

(5) $$Ln\left(q_{e}\right)=\frac{1}{n}Ln\left(C_{e}\right)+Ln\left(K_{F}\right)$$

where: C_e_ and q_e_ are defined as before; K_F_=Freundlich constant related to the adsorption capacity of the adsorbent (mg/g) and the adsorption strength of the solute $\left(\frac{{mg}^{\left(1-\frac{1}{n}\right)}L^{\frac{1}{n}}}{g}\right)$ ; and n=Freundlich exponent related to the heterogeneity of the surface (adsorption intensity) of the adsorbent and the adsorption strength of the solute (L/mg).

The Freundlich exponent n is a measure of the deviation from linearity of the adsorption isotherm, with values of n between 1 and 10 indicating favorable adsorption conditions, values of n greater than 10 indicating very favorable adsorption conditions, and values of n less than 1 indicating less favorable adsorption conditions.

The equilibrium data with Temkin isotherm model is given in Eq. 6.

(6)

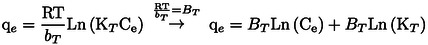




Where C_e_ and q_e_ are defined as before, K_T_=Temkin isotherm equilibrium binding constant $\left(\frac{L}{g}\right),\;$ b_T_=Temkin isotherm constant, R=universal gas constant, T=Temperature, B=Constant related to heat of sorption $\left(\frac{J}{mol}\right)$ . The constants are determined by the regression analysis of linear plots q_e_ verses Ln(C_e_)

***Kinetic studies. Lagergren pseudo‐first order rate equation***. The Lagergren pseudo‐first order rate equation is a commonly used model to describe the adsorption kinetics of a solute onto a solid surface. The equation is based on the assumption that the rate‐limiting step of the adsorption process is the transfer of the solute from the bulk solution to the surface of the adsorbent (Eq. 7.

(7) $$Ln\left(q_{e}-q_{t}\right)=-k_{1}t+Ln\left(q_{e}\right)$$

Where; q_e_ is defined as before, q_t_=the amount of solute adsorbed per unit mass of adsorbent (mg/g) at a time t, *k*_1_=first order rate constant of adsorption,

The Lagergren pseudo‐first order rate equation assumes that the rate of adsorption is directly proportional to the number of unoccupied sites available on the surface of the adsorbent. It also assumes that the concentration of the solute in the bulk solution remains constant throughout the adsorption process.

The pseudo‐second‐order model (Eq. 8 is another commonly used model to describe the adsorption kinetics of a solute onto a solid surface.

(8) $$q_{t}=\frac{k_{2}\cdot q_{e}^{2}\cdot t}{1+k_{2}\cdot q_{e}\cdot t}\to \frac{1}{q_{t}}=\frac{1}{k_{2}\cdot q_{e}^{2}\cdot t}+\frac{1}{q_{e}}\to \frac{t}{q_{t}}=\left(\frac{1}{q_{e}}\right)t+\frac{1}{k_{2}\cdot q_{e}^{2}}$$

where, q_e_, q_t_, and t are as before and k_2_ is the second‐order rate constant of adsorption $\left(\frac{g}{mg\cdot s}\right)$ .

The model assumes that the rate determining step of the adsorption process is chemisorption, which involves valency forces through coordination or exchange of electrons between the adsorbent (BCFO) and the adsorbate (ARS dye). The pseudo‐second‐order model assumes that the rate of adsorption is proportional to the square of the number of unoccupied sites available on the surface of the adsorbent. This model can provide a better fit to experimental data in some cases, especially when the adsorption process is dominated by chemisorption.

***Quantum chemical calculations***. All DFT calculations were performed with the Amsterdam Density Functional (ADF) program^[13]^ using relativistic, dispersion‐corrected density functional theory (DFT) at the ZORA‐B3LYP‐D3(BJ)/TZ2P level of theory^[14]^ for geometry optimizations and energy calculations,^[15]^ with the full electron model for all atoms (no frozen core), *in vacuo*. All stationary points were verified to be minima on the potential energy surface through vibrational analysis. TD‐DFT calculations^[16]^ were carried out at the CAMY‐B3LYP/TZ2P level.

## Results and Discussion

Once brownmillerite type Ca_2_Fe_2_O_5_ nanoparticles were prepared, this catalyst was characterized with the techniques described above (see also Methodology section for further details). And next we will focus on the main contribution of this work, that involves the degradation of ARS quinone dye with the new proposed method, i. e., in the absence of. any acid or base additives and under darkness conditions.

### X‐ray Diffraction Analysis

**Figure 2:**
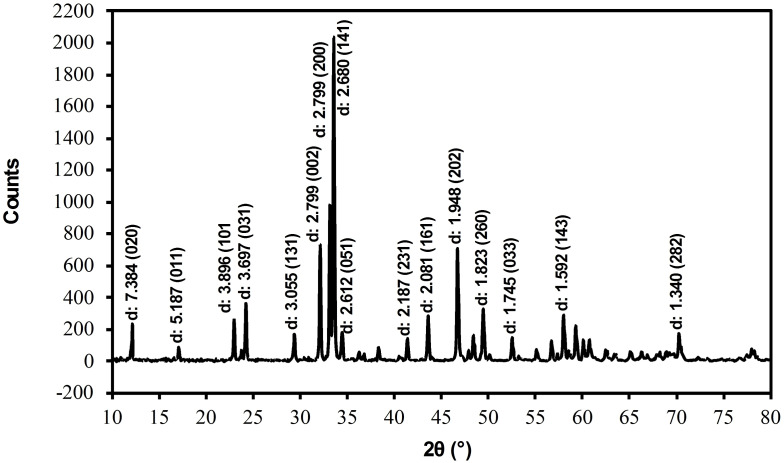
X‐ray diffraction pattern of BCFO sample. d spacings are in Å.

X‐ray diffraction (XRD) patterns are used to characterize the prepared BCFO sample (Figure 2).^[17]^ The data was investigated using the commercial X'Pert High Score package. All diffraction peaks of X‐ray pair nicely with those of the reported X‐pert high score PDF code 01‐071‐2264. Results of XRD analysis indicates the foundation of an orthorhombic Ca_2_Fe_2_O_5_ structure with the cell parameters obtained: a=5.43 Å, b=14.77 Å, and c=5.60 Å, with space group *Pnma*. The average crystallite size of 70 nm is estimated using the Debye‐Scherrer formula concerning Bragg reflection at intense peaks of (002), (200), (141) and (202).

### Fourier Transform Infrared Spectroscopy

**Figure 3:**
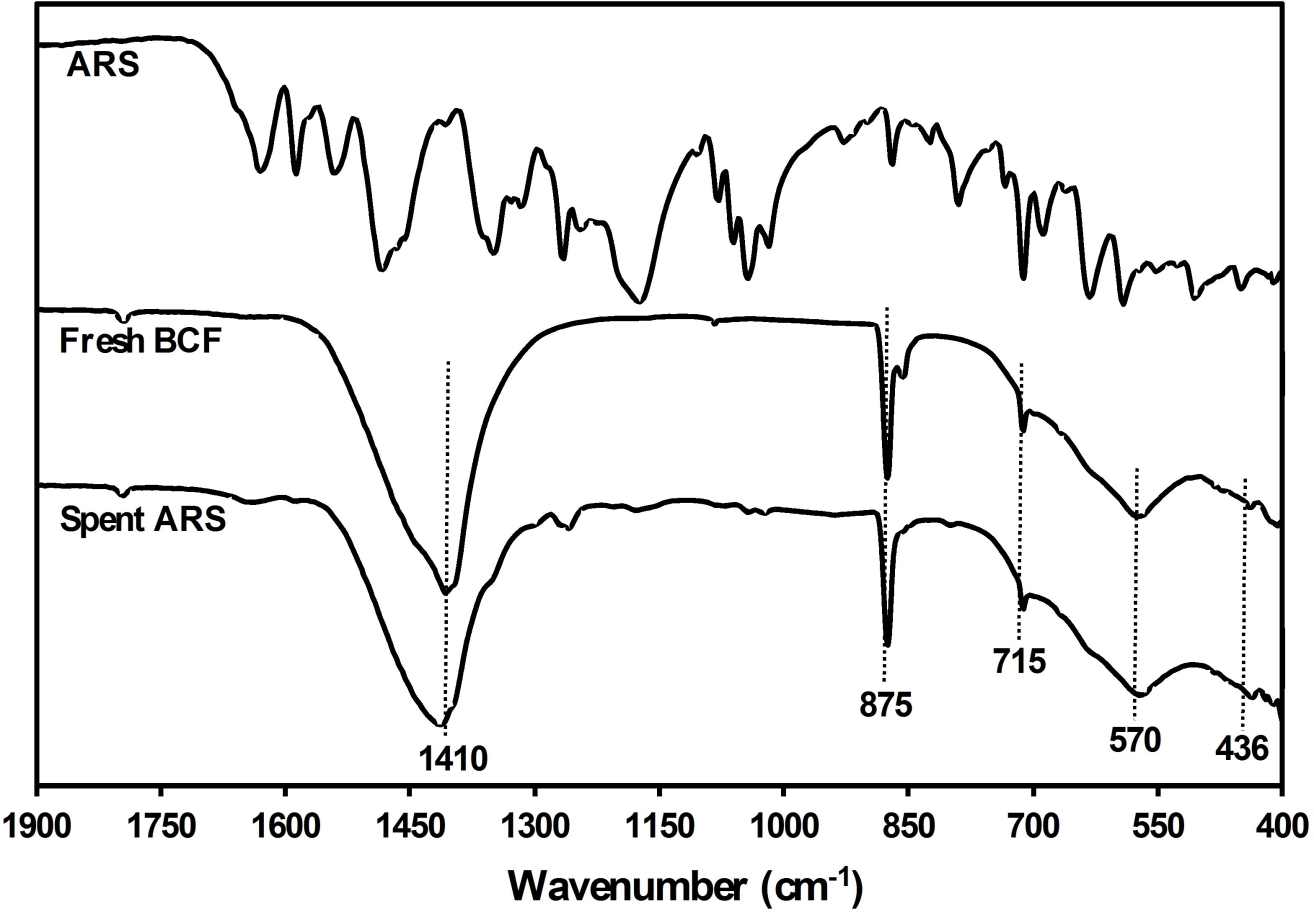
Fourier Transform Infrared spectra of the ARS, fresh and spent BCFO perovskite‐related specimens.

Figure 3 displays the Fourier Transform Infrared (FTIR) spectra recorded for the ARS, fresh and spent BCFO perovskite‐related specimens. A same infrared spectrum as the freshly prepared sample is observed for the used catalyst after the catalytic degradation. The bands below 800 cm^−1^ in the FTIR spectrum of the brownmillerite structure are assigned to the stretching vibration of the metal‐oxygen bond. The wide band centered at 571 cm^−1^ and the smaller band at 436 cm^−1^ correspond to the Fe−O bond stretching in tetrahedral sites. A peak at 715 cm^−1^ is attributed to the Ca−O bond stretching. The bands at 1415 and 1484 cm^−1^ and weak peaks at 875 and 858 cm^−1^ are attributed to the metal carbonate vibrations, due to the formation of carbonate species on the oxide surface.[^6^, ^18^] Almost no carbonate peak was observed in the XRD analysis, indicating a low concentration of this species, and thus it is not at the level of the detection limit of this analysis.

The same infrared spectrum for the fresh catalyst and the used one after the catalytic degradation shows that following the adsorption, the catalytic degradation continues even under darkness. In other words, it confirms the catalytic degradation over the BFCO catalyst. However, further discussion on degradation will be developed below.

### Compositional and Morphological Studies

The energy dispersive spectrum studies of the solution mixture‐synthesized BCFO reveal that the resulting nanoparticles are primarily composed of Ca, Fe, and O elements (Figure S1 in the Supporting Information). The data of the EDS analysis enclosed in Table 1 confirms that the expected composition of BCFO has been successfully achieved, thus supporting the Ca_2_Fe_2_O_5_ ⋅ 2H_2_O formula. These findings confirm that the chosen reaction pathway was appropriate for this synthesis.

**Table 1:** Results of Energy Dispersive Spectroscopy (EDS) spectra for synthesized BCFO.

	Experimental		Theoretical/Experimental
Element	Wt (%)	Atomic (%)	
O	36.83	63.58	Ca_2_Fe_2_O_5_/Ca_2.034_Fe_2_O_5.034_ ⋅ 2H_2_O
Ca	26.65	18.37	
Fe	36.51	18.06	Ca_2.034_Fe_2_O_7.034_/Ca_2.034_Fe_2_O_5.034_ ⋅ 2H_2_O
Total	99.99	100.01	

**Figure 4:**
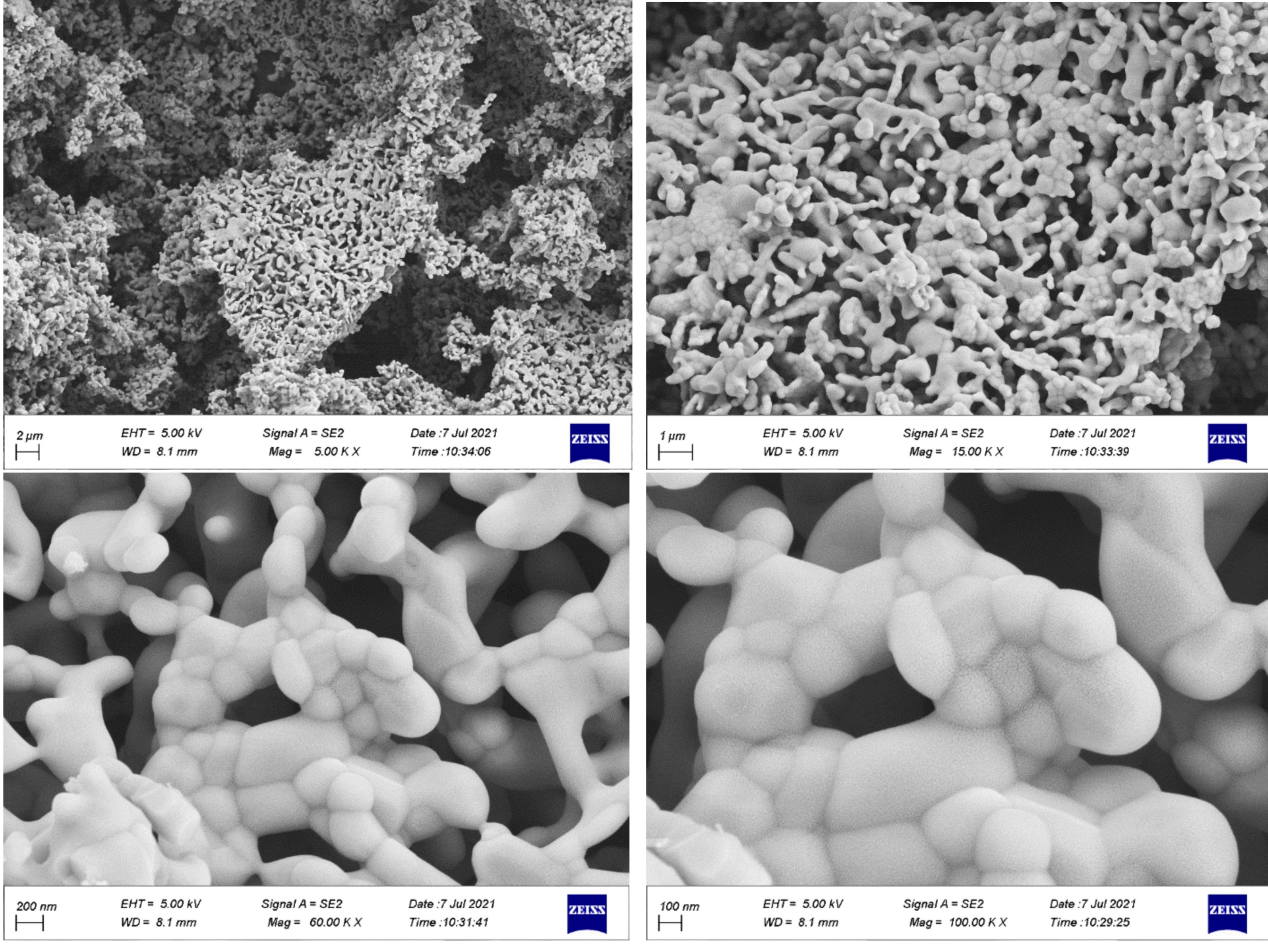
Field Emission Scanning Electron Microscopy (FESEM) images of BCFO sample.

Figure 4 allows to examine the surface morphology of the Ca_2_Fe_2_O_5_ nanoparticle by analyzing the Field Emission Scanning Electron Microscopy (FESEM) images. The samples exhibit a sponge‐like structure without any amorphous or crystallized phases other than BCFO. The porous morphology of Ca_2_Fe_2_O_5_ is evident from the large interspaces between particles, as illustrated in Figure 4. This porous structure is believed to play a crucial role in catalyzing the degradation of ARS dye. When the solute molecules come into contact with the porous surface of the adsorbent, the adsorption process is amplified. This strengthening occurs because a chemical reaction takes place in the proximity of the catalyst, mediated by the contact between the solute molecules and the adsorbent surface. It is worth noting that the synthesis method can influence the shape and structure of the particles, as seen in the FESEM images. This may explain why proper degradation of ARS dye was achieved in this study.

### UV‐Vis Spectra of ARS

**Figure 5:**
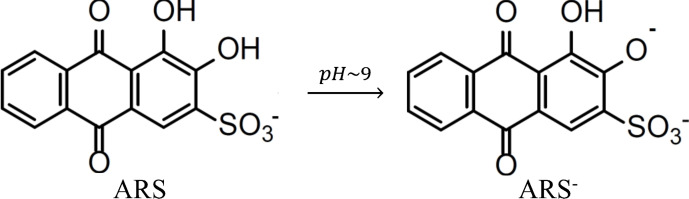
Transforming ARS into the form of ARS^−^ under basic pH conditions.

Alizarin Red S is formed with the introduction of a sulphonate group in the alizarin structure in position 3 (Figure 5). It consists of an anthraquinonic dye whose sulfonic acid group induces its solubility in water. As the pH of the initial ARS aqueous solution increases, the faint yellow color (at pH ~6) changes to a light pink hue (at pH ~9). The compound ARS has two enolic groups capable of ionization (at positions 1 and 2), which occurs in a basic medium (Figure 5).^[19]^ A similar behavior can be assigned when BCFO powder is added to a solution of ARS in water. The results suggest that the use of BCFO catalyst drives to the production of monoprotic ARS species, that is, BCFO deprotonates ARS.

**Figure 6:**
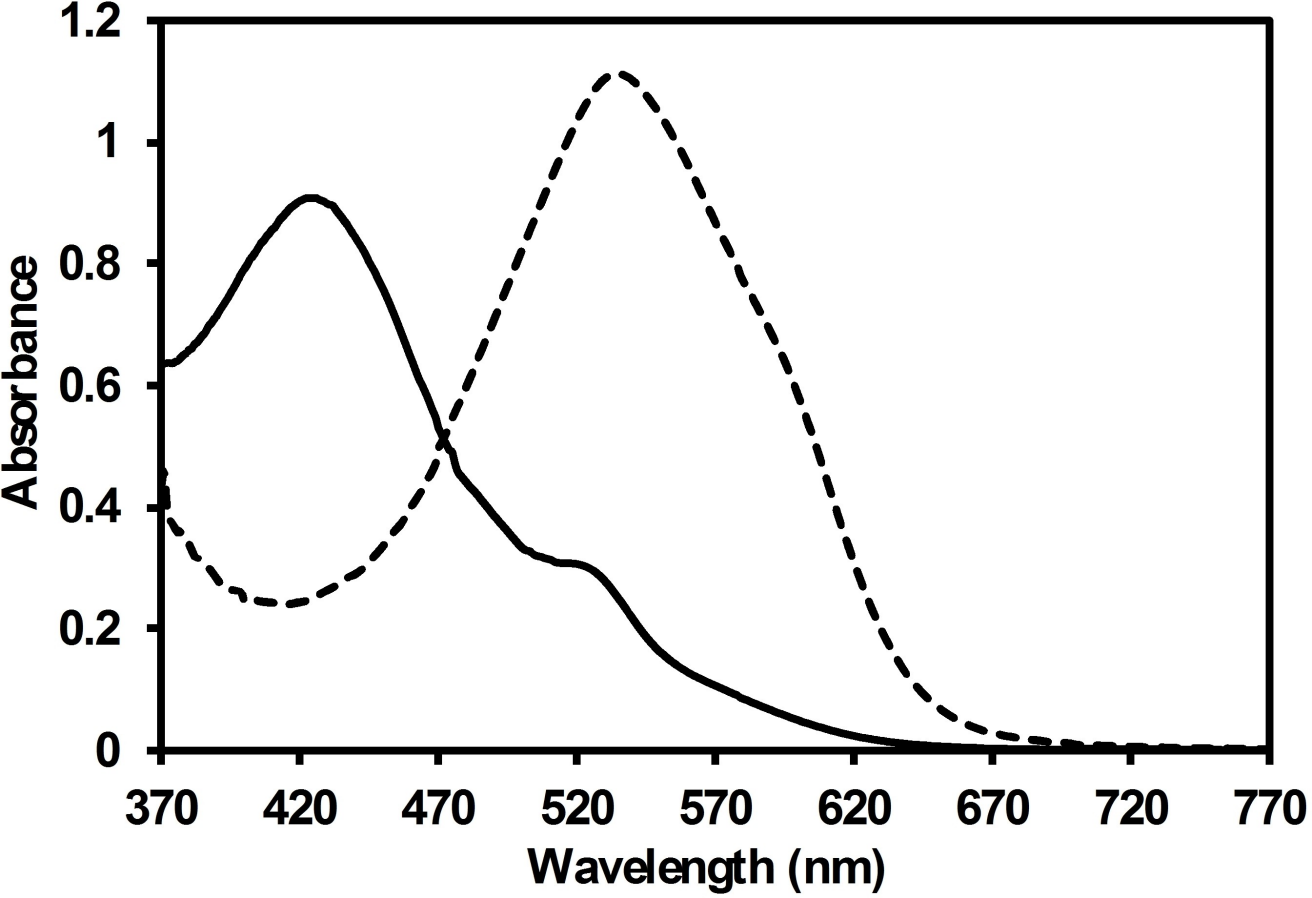
Absorption spectra of aqueous solution of 100 ppm ARS in the absence (solid line) and presence (discontinuous line) of 10 mg BCFO sample. The spectrum was recorded 5 min after the addition of the catalyst.

ARS has two chromophores, that is, OH groups,^[20]^ that react with the basic surface of BCFO. In the obtained absorption spectrum of ARS we observe a peak wavelength at 425 nm (Figure 6), which is red‐shifted to 535 nm when BCFO is added (visible spectrum). Thus, the above bathochromic shift of 110 nm, that is,., from 425 to 535 nm, is attributed to the formation of the mono‐protic ARS species (ARS^−^) and is due to the extended conjugation of this species. With the aim to get further insight into the UV‐Vis data, a quantum chemical analysis has been performed below.

**Figure 7:**
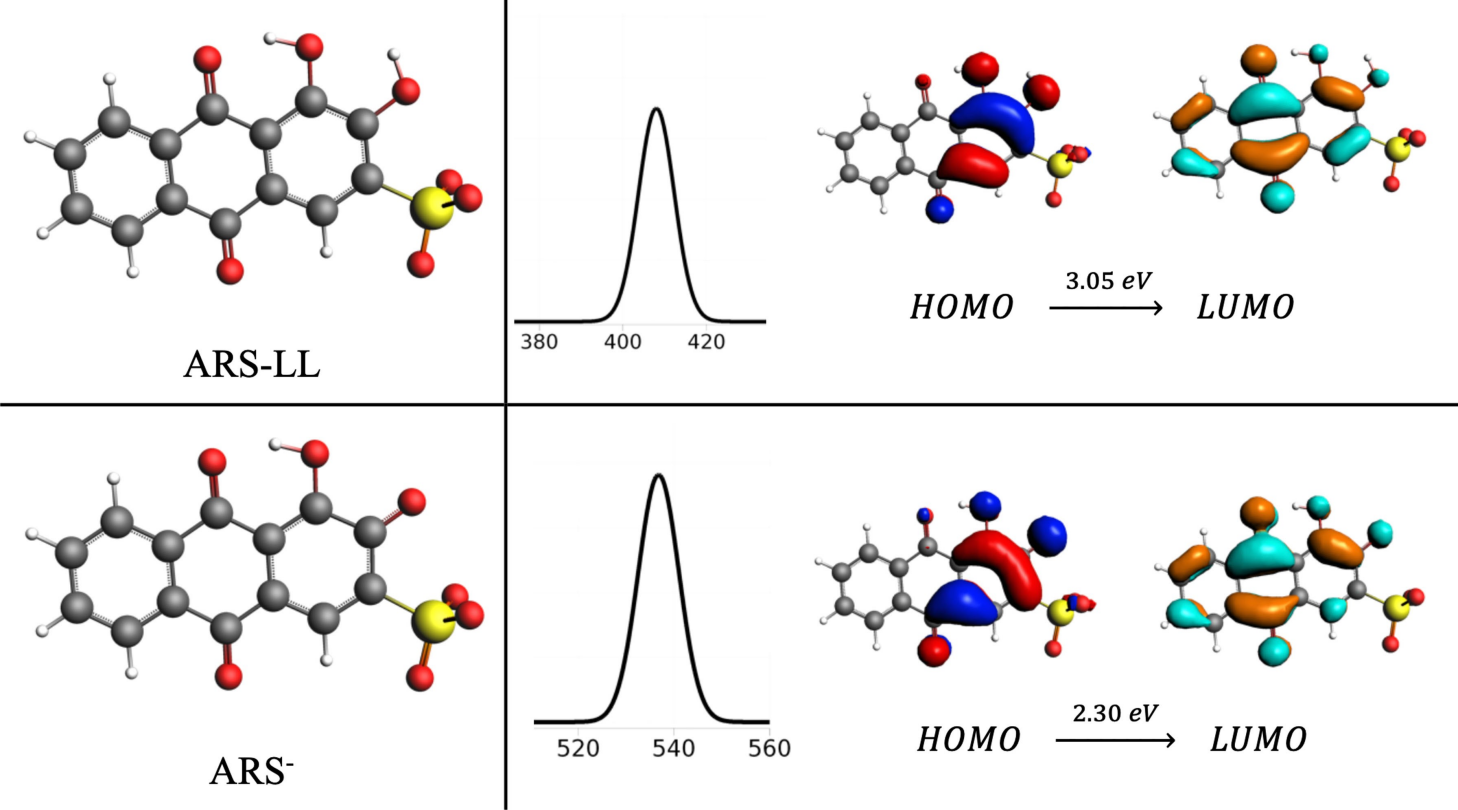
Structure, UV‐Vis spectra band, and HOMO and LUMO π molecular orbitals of ARS and its monoprotic ion. HOMO → LUMO band at 407 and 536 nm for ARS and ARS^−^, respectively. HOMO‐LUMO gap is 5.30 and 4.46 eV for ARS and ARS^−^, respectively.

Next, a time‐dependent density functional theory analysis was conducted in water at the CAMY−B3LYP/TZP level of theory for both ARS and ARS^−^ species (Figure 7). We observe a good agreement with experimental spectrum above.^[21]^ In particular, ARS presents a HOMO→LUMO peak at 415 nm (average of the three possible isomers), which is red‐shifted to 535 nm when the proton is removed. Importantly, this peak corresponds to a π→π* transition in both cases. The HOMO‐LUMO gap is reduced from ARS to ARS^−^ (from 5.30 to 4.46 eV), due to a less stable π HOMO orbital (Tables S1 and S2 in the Supporting Information).

### CO_2_ Temperature Programmed Desorption Study

The electronic absorption spectrum and computational studies above point out that the catalyst has a moderately basic surface. To further investigate the basic nature of the catalyst surface, a CO_2_‐Temperature Programmed Desorption **(**TPD) study was conducted. Thus, with the aim to identify the basic sites of the catalyst, the BCFO underwent a CO_2_‐TPD analysis.

**Figure 8:**
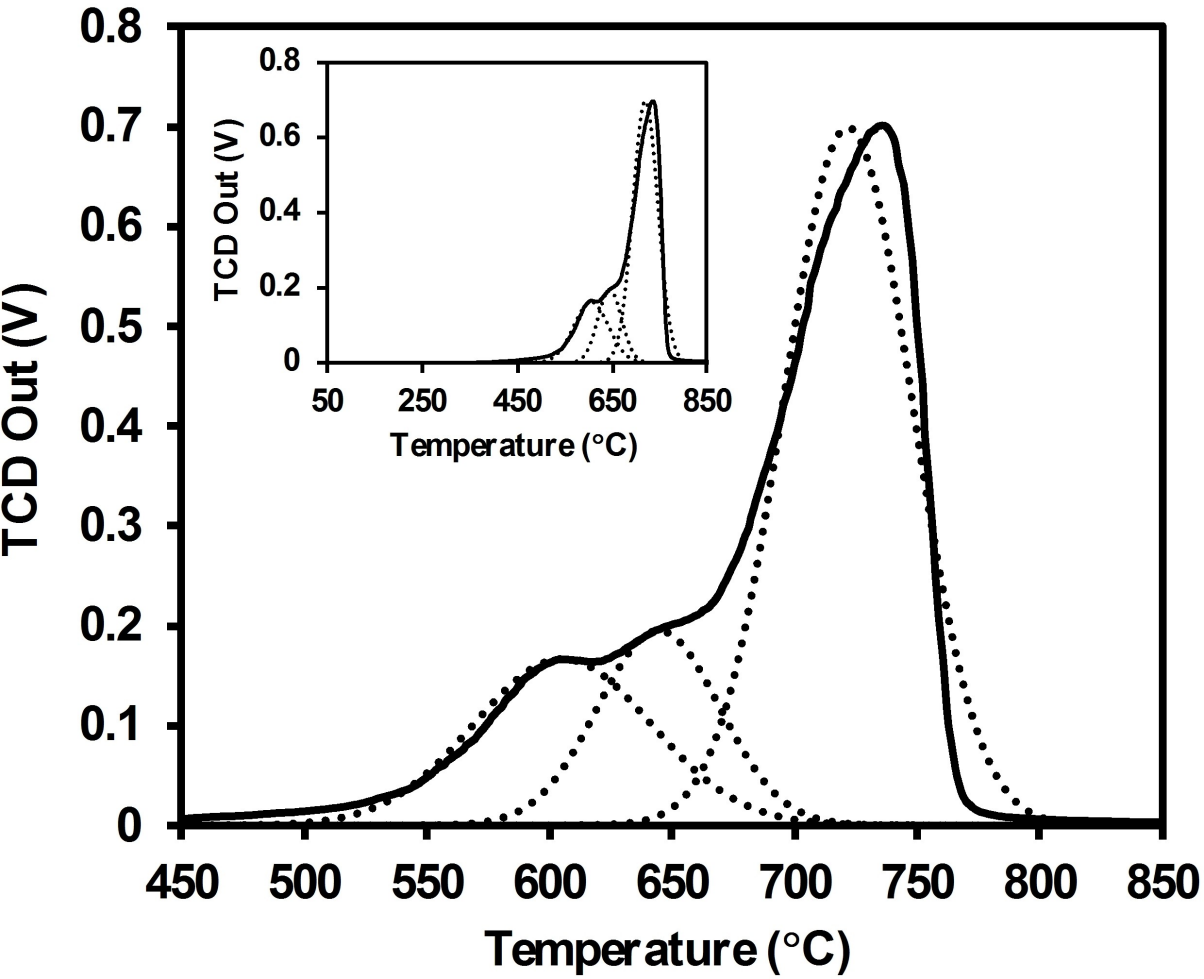
CO_2_‐TPD profiles of synthesized BCFO catalyst.

Figure 8 displays the carbon dioxide TPD profile of the synthesized BCFO sample in the temperature range of 450–850 °C. The broad CO_2_‐TPD profile is a result of the overlapping peaks. To determine the number of basic sites with different strengths, a semi‐quantitative comparison of basicity was carried out using Gaussian deconvolution of the TPD profiles. The total basicity and distribution of basic sites were obtained from the areas under the peaks and their positions in the TPD profile, respectively. The primary CO_2_‐TPD peaks of the BCFO are located at approximately 500–800 °C. Based on the temperature of peak maxima at approximately 605, 645, and 715 °C, the Gaussian curves can be categorized as three types of weak, medium, and strong basic sites, respectively. However, due to the proximity of the peaks, there is only a slight difference in basic strength between the first two sites. Table 2 presents the estimated amount and strength of basicity in the sample obtained from CO_2_‐TPD, indicating the presence of predominantly strong basic sites.

**Table 2:** Basic properties of synthesized BCFO catalyst.

Basicity (μmol CO_2_/g‐catalyst)			
Weak basic sites	Medium basic sites	Strong basic sites	Total basic sites
540 (20 %)	459 (17 %)	1701 (63 %)	2700

Metal oxides are composed of metal cations and oxide ions on their surface. Nano BCFO is recognized as a stable material that can quickly capture (carbonation) and release (de‐carbonation) CO_2_.^[22]^ Calcium oxide has also been subjected to a comparable examination of its CO_2_‐TPD profile.^[23]^ Studies have been conducted on the adsorption of CO_2_ by perovskite and CaO‐based oxides within the temperature range of 600 °C to 900 °C.^[24]^ Carbon dioxide can stably chemisorb on the surface of the BCFO catalyst.

### Physico‐Chemical Properties

**Figure 9:**
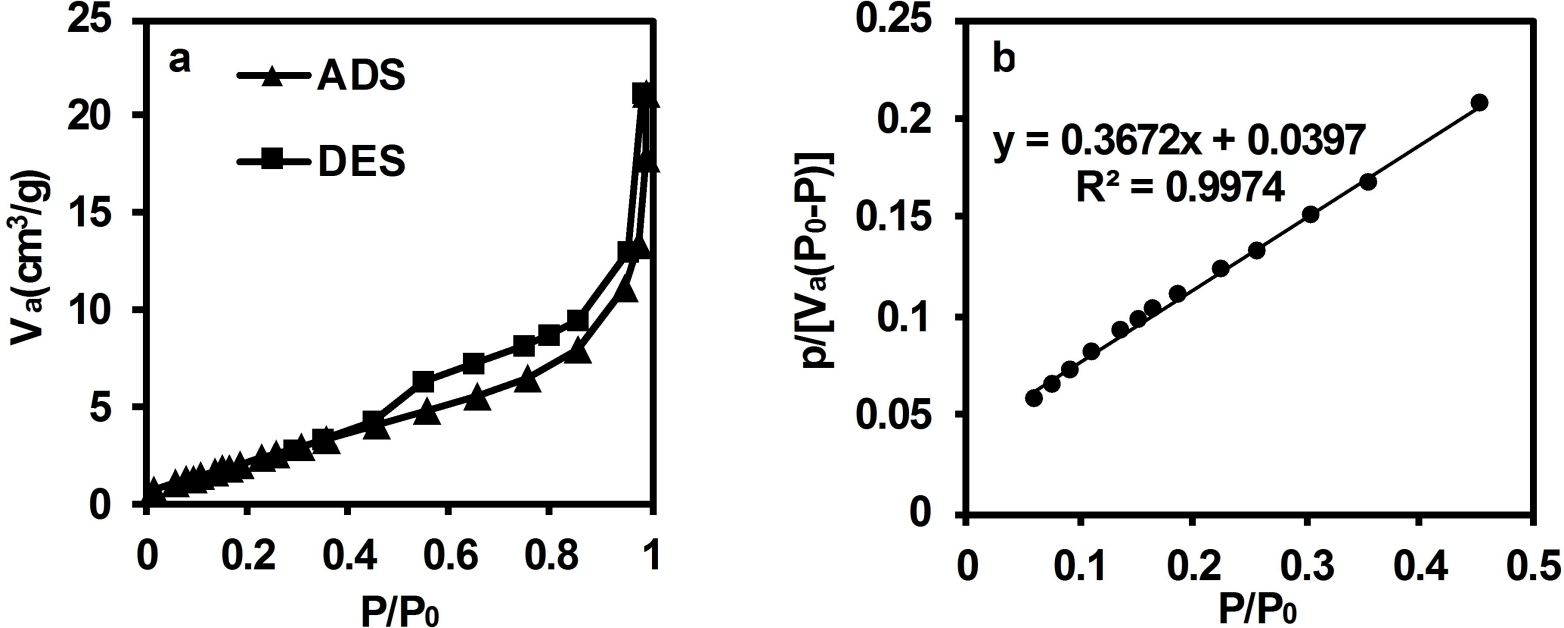
a) Hysteresis loop of the N_2_ adsorption‐desorption and b) BET plot (SD=0.0056 and 0.0013 for slop and intercept, respectively).

The quantification of Brunauer‐Emmett‐Teller (BET) surface area, which is a vital parameter for characterizing solid materials, is typically done by analyzing nitrogen adsorption isotherms measured at 77 K using the Brunauer, Emmett, and Teller theory. Originally developed to describe multi‐layer gas adsorption on surfaces, this parameter is now widely used to report the surface area of materials.^[25]^ In this study, the BET technique was employed to determine the specific surface area and average pore size of BCFO. Figure 9 depicts the nitrogen adsorption‐desorption isotherms of the BCFO sample and the corresponding BET plot.

A hysteresis loop of type H3 is observed in the adsorption‐desorption isotherm. This type of loop is identified by the lower limit of the desorption branch, which is typically located at the p/p_0_ caused by cavitation.^[26]^ Flexible assemblies of plate‐shaped particles and pore networks that contain unsaturated macro‐pores often exhibit such loops. The Barrett‐Joynere‐Halenda (BJH) desorption theoretical model was used to determine the pore volume (Table 3), which is a useful technique for analyzing the pore size distribution in a porous material. The porosity of BCFO was also confirmed through FESEM analysis above (Figure 2).

**Table 3:** BET, pore volume, and pore diameter data of synthesized BCFO sample.

BET $\left(\frac{m^{2}}{g}\right)$	Total pore volume $\left(\frac{{cm}^{3}}{g}\right)$	Mean pore diameter (nm)
10.00	0.034	1.21

### BCFO Bandgap Estimation

**Figure 10:**
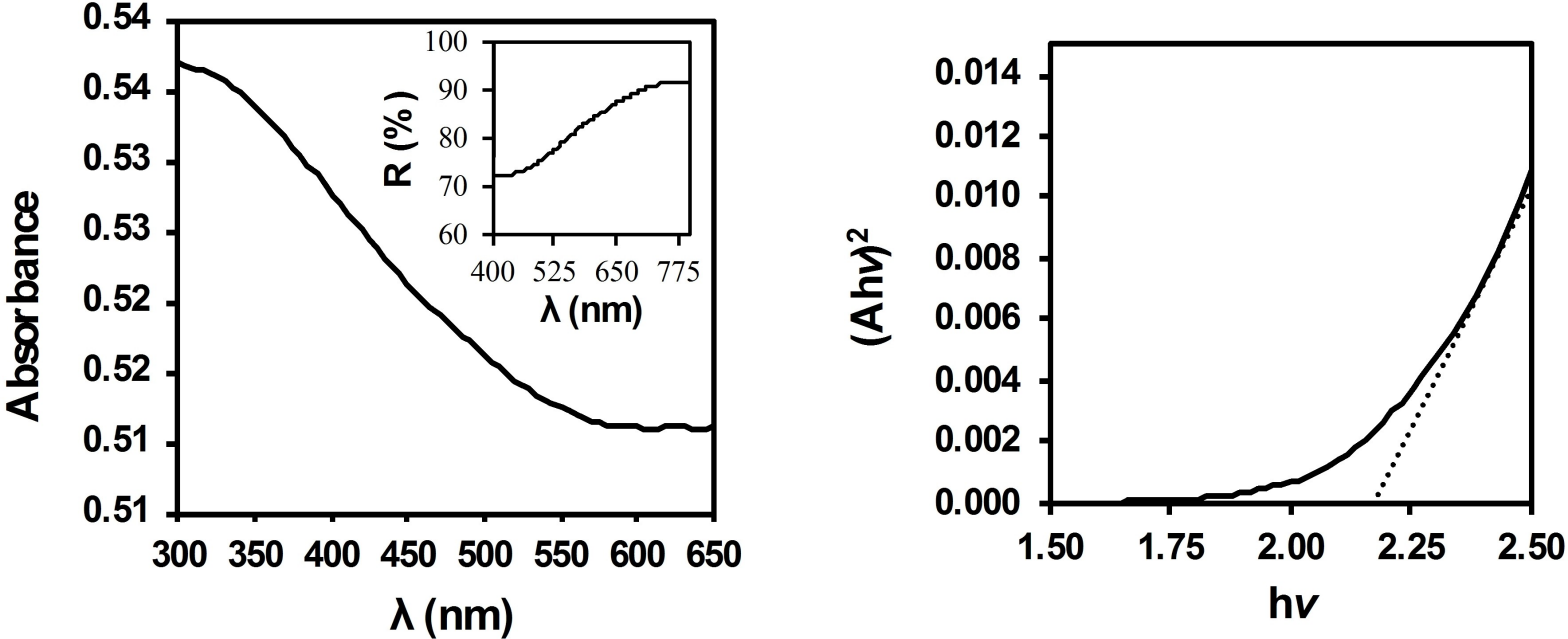
UV‐Vis absorption spectra of BCFO nanoparticles (left), and graph of Tauc's equation used to determine the band gap of BCFO nanoparticles (right). The inset is the DR‐UV‐Vis spectra of the solid sample.

The semiconductor‘s total energy difference between the HOMO and LUMO can be roughly estimated using the bandgap.^[27]^ This bandgap in BCFO has been estimated using the UV‐Vis absorption edge studies. The DR‐UV‐Vis spectrum of BCFO nanoparticles exhibits significant absorption in the almost end of the visible region. It was converted to the A/λ plot and Tauc equation (see the Materials and Methods section) to calculate the band gap energy (Figure 10).[^17^, ^28^]

The Tauc equation is often plotted as a linear function of (Ahν)^2^ vs. hν, which allows for the determination of the bandgap energy, Eg, from the intercept of the linear fit. By extrapolating the linear portion of the (Ahυ)^2^ against hυ plot to point A=0, the estimated band gap energy of BCFO is approximately 2.17 eV (Figure 10). It is also supported by the absorption edge of BCFO, which is observed to be at about 550 nm (2.25 eV). Results show that the BCFO sample seems to have photocatalytic ability in the visible region. To prove the high novelty of the prepared brownmillerite, however, its catalytic activity in the degradation of ARS dye has been carried out in the absence of any acid or base additives, and under darkness conditions.

### XPS Spectra

**Figure 11:**
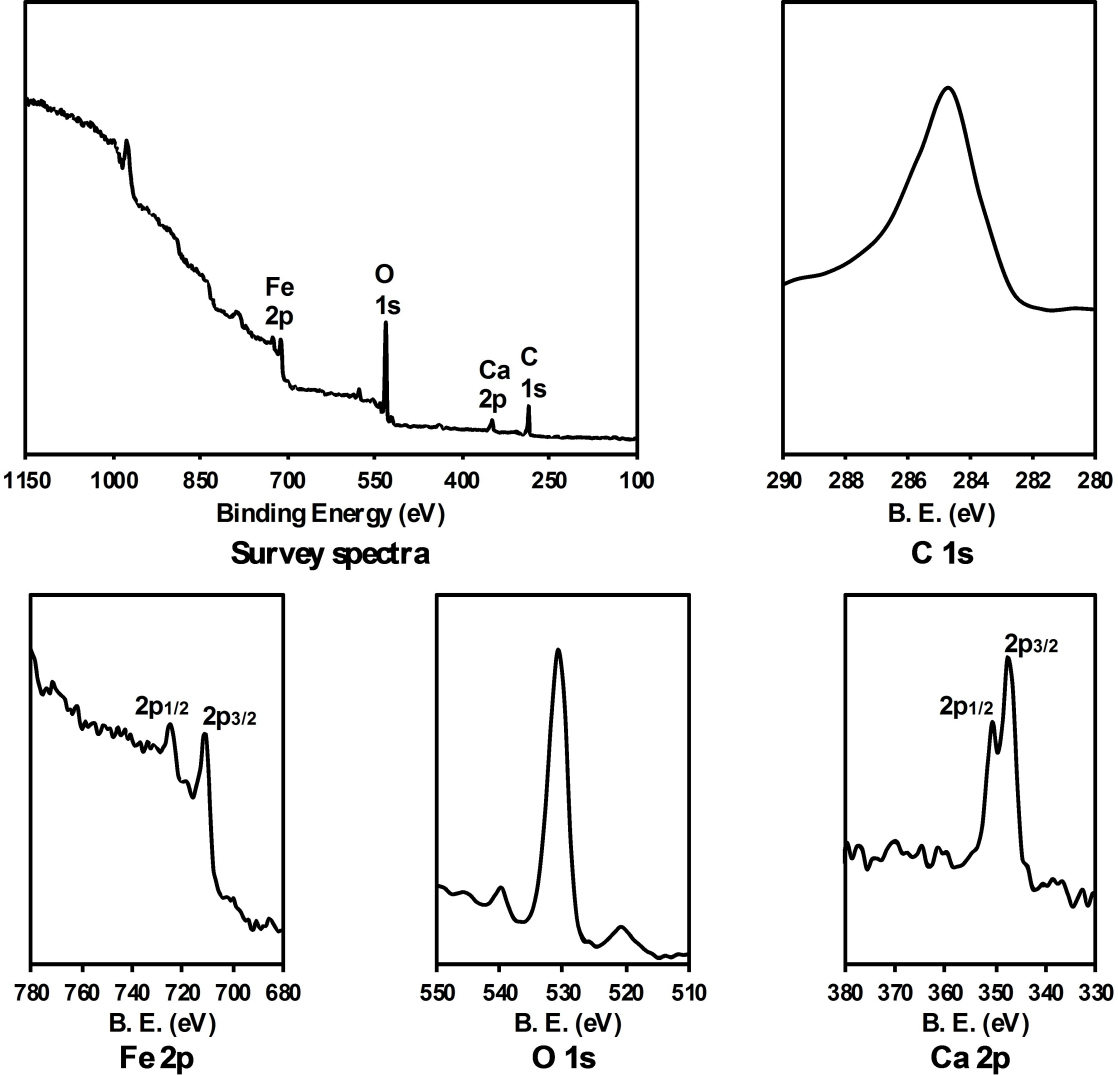
XPS features of the BCFO nanoparticles in their as‐prepared state in the regions of the Fe, O and Ca core levels. The binding energy for Fe corresponds to peaks at 724 and 711 eV. The O 1s core level spectra consist of a component at 531 eV. The binding energy for Ca correspond to peaks at 350 and 347 eV.

X‐ray photoelectron spectroscopy (XPS) measurements were performed to confirm the stoichiometry and the valence states of constituent elements in the bulk BCFO sample (Figure 11). The XPS peaks are referenced to the C 1s binding energy, which is located at 285 eV. The identification of chemical states is crucial, and in the case of oxidized iron, it relies on the presence of satellite peaks accompanying the iron 2p photoelectron peaks. When examining XPS spectra for Fe ions, a noticeable shift towards higher binding energy is observed in the Fe oxide peaks compared to those of the metal. The Fe 2p_3/2_ peak is located at a binding energy of 711 eV, accompanied by a weakly present satellite peak 6 eV above, which corresponds to the Fe^3+^ 2p_3/2_ state. Additionally, the peak at a binding energy of 724 eV with a corresponding satellite peak around 733 eV indicates the presence of Fe(III) in the 2p_1/2_ state. It supports the conclusion that Fe exists in the (3+) form. The XPS peak for O 1s lies in the binding energy of 530 eV. It is reported that a broadening O 1s binding energy may be attributed to multiple overlapping components, like adsorbed water and carbonate at the surface.^[29]^ The straightforward interpretation of the O 1s spectra for this mixed metal oxide is supported by the presence of a peak at 531 eV, which can be attributed to O within the lattice. Finally, the peaks at 347 and 350 eV are attributed to Ca 2p_3/2_ and 2p_1/2_, respectively. This suggests that Ca exists in (2+) state. Weak satellite loss features were observed in the Ca 2p region at 364 eV. Results of the XPS study are fully consistent with the EDX analysis.

### Degradation Studies

The decomposition of dyes that are difficult to degrade causes a great interest not only in water treatment but also in several industrial applications. The anthraquinone dyes, like ARS, are recognized as persistent dyes that undergo minimal degradation in the environment. The discovery of an inexpensive and potent catalyst for this purpose, particularly under regular circumstances, is crucial. Results of this study have demonstrated that the BCFO is a promising material for this objective. As already pointed out above, the infrared spectrum for the fresh catalyst and the used one after the catalytic degradation (Figure 3) shows that following the adsorption, the catalytic degradation continues even under darkness.

The total organic carbon (TOC) and IC analyses were carried out to further confirm this degradation.[^19^, ^28c^] The corresponding results are enclosed in Table 4 for the fresh and degraded ARS solutions. The decrease of TOC greater than 60 % confirms such degradation. In addition, the large increase in the IC shows that the degradation persists with to the closing stage, that is, the formation of carbon dioxide.

**Table 4:** TOC and IC analyses results in ppm. Results are for 10 mL of a 100 ppm solution.

ARS sample	TOC $\left(\frac{mg\;C}{l}\right)$	IC $\left(\frac{mg\;C}{l}\right)$
Fresh (no catalyst)	34.2	0.6
Spent (in the presence of catalyst, degraded)	12.2	11.6

**Figure 12:**
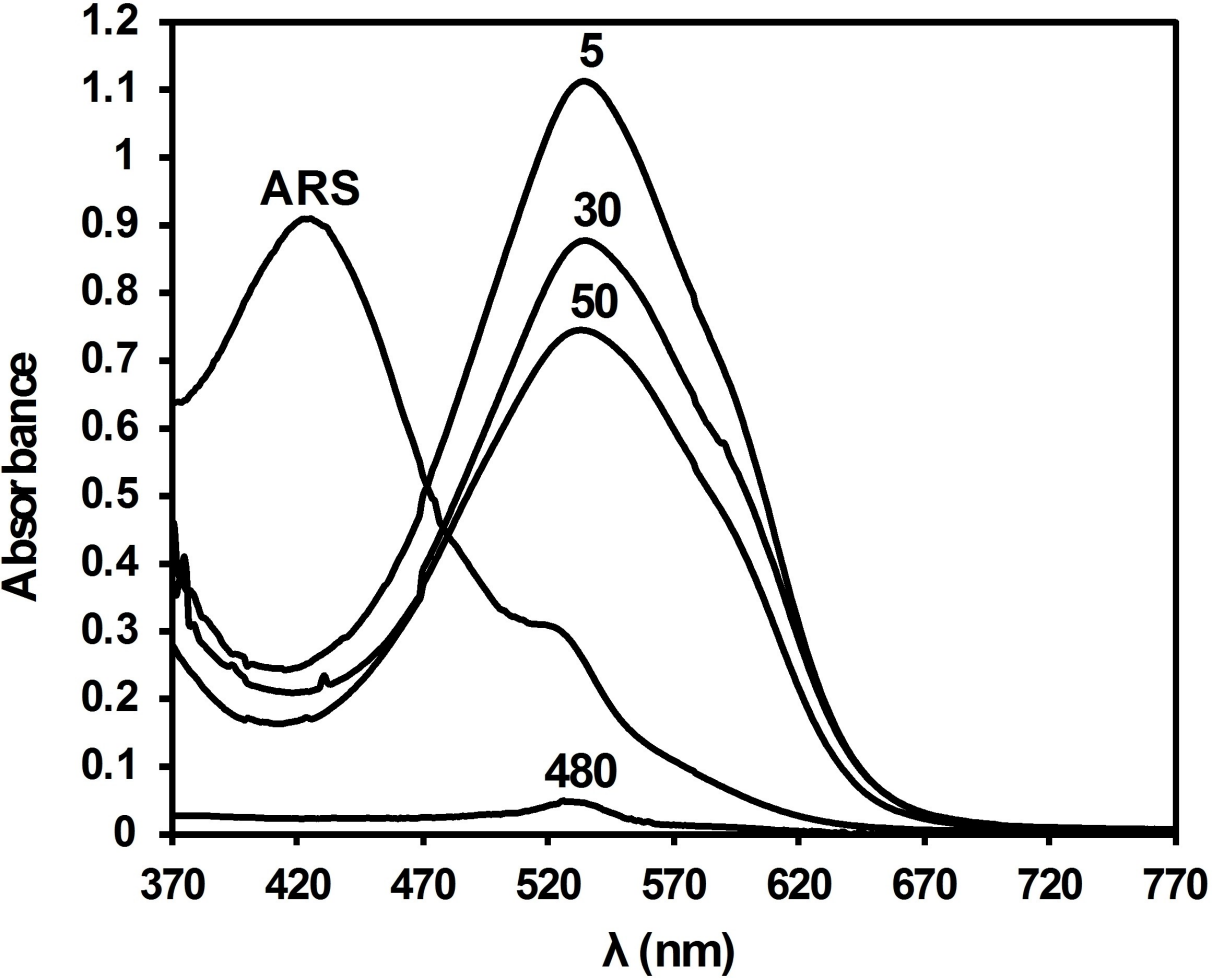
Electronic absorption spectroscopy results of 100 ppm aqueous ARS solution in the absence and presence of BCFO at different time intervals. Numbers are in minutes.

Next, we observe how under dark ambient conditions and without the presence of any acidic or basic additives, a solution comprising 100 ppm of ARS in water and 10 mg of BCFO catalyst decomposes by more than 70 % in about 2 h (Figure 12 and Figure S2 in the Supporting Information). This result shows that the BCFO is a potent and promising catalyst for this kind of hard‐degradable dyes. The enhancement of catalytic activity is being investigated by exploring the optimization of the catalyst‘s surface area and basicity, and it is expected to give rise to an increased efficiency, although further research is still ongoing. For completeness, the degradation of ARS using BCFO was further studied using isothermal and kinetic modeling.

### Isothermal Modeling of Equilibrium Data

**Figure 13:**
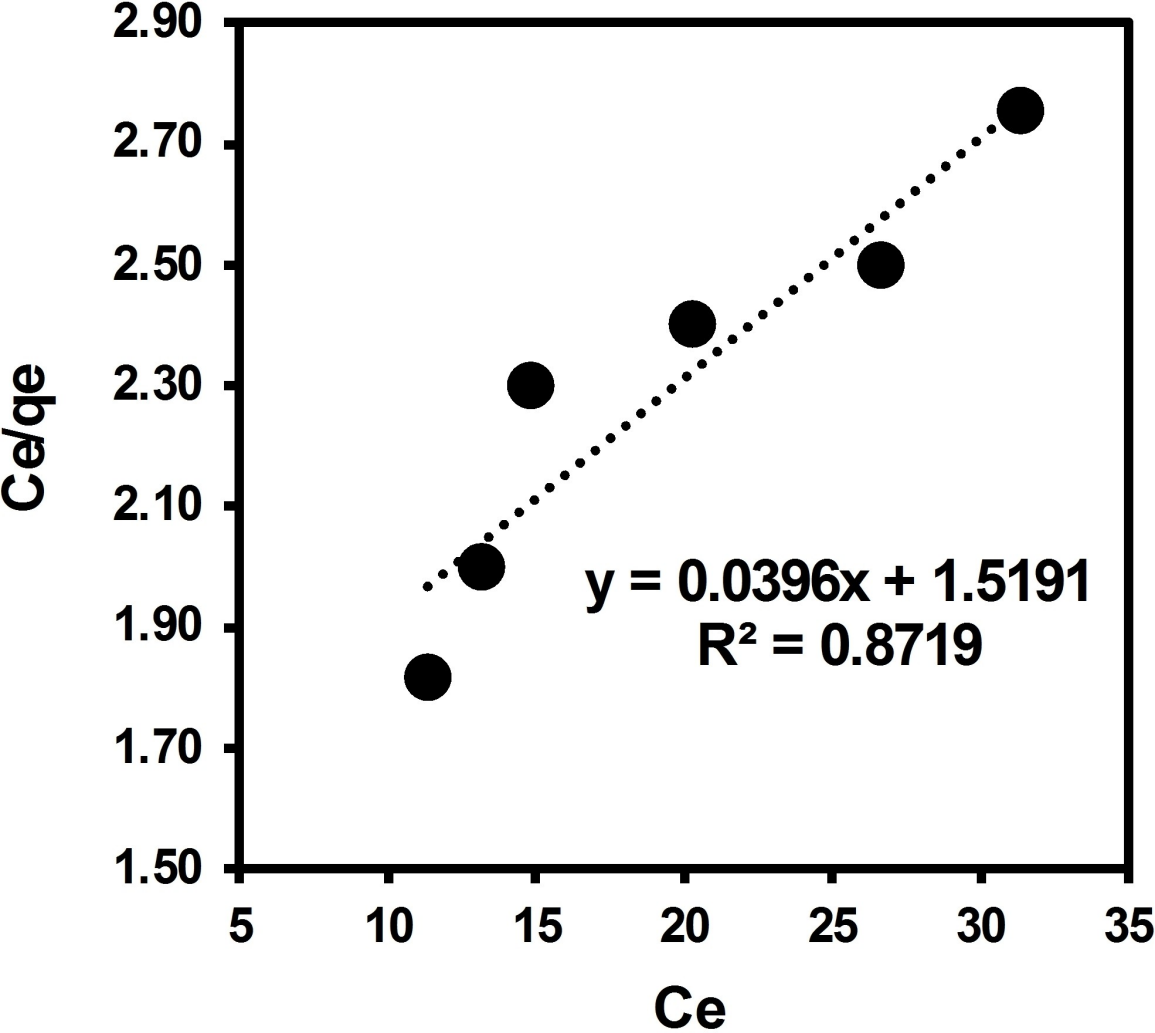
Langmuir isotherm for adsorption of ARS dye over BCFO in solution.

**Figure 14:**
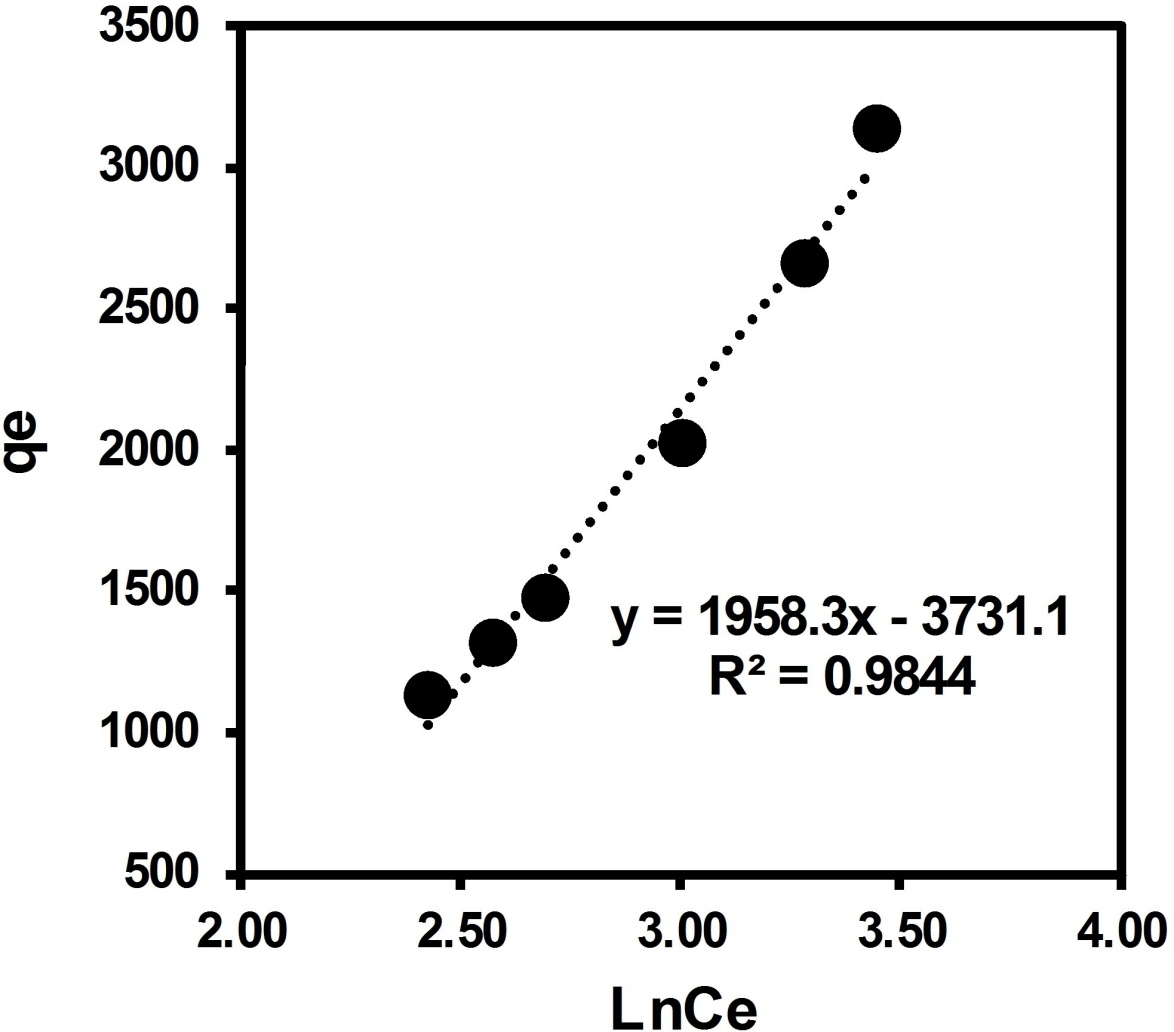
Temkin isotherm for adsorption of ARS dye over BCFO.

**Figure 15:**
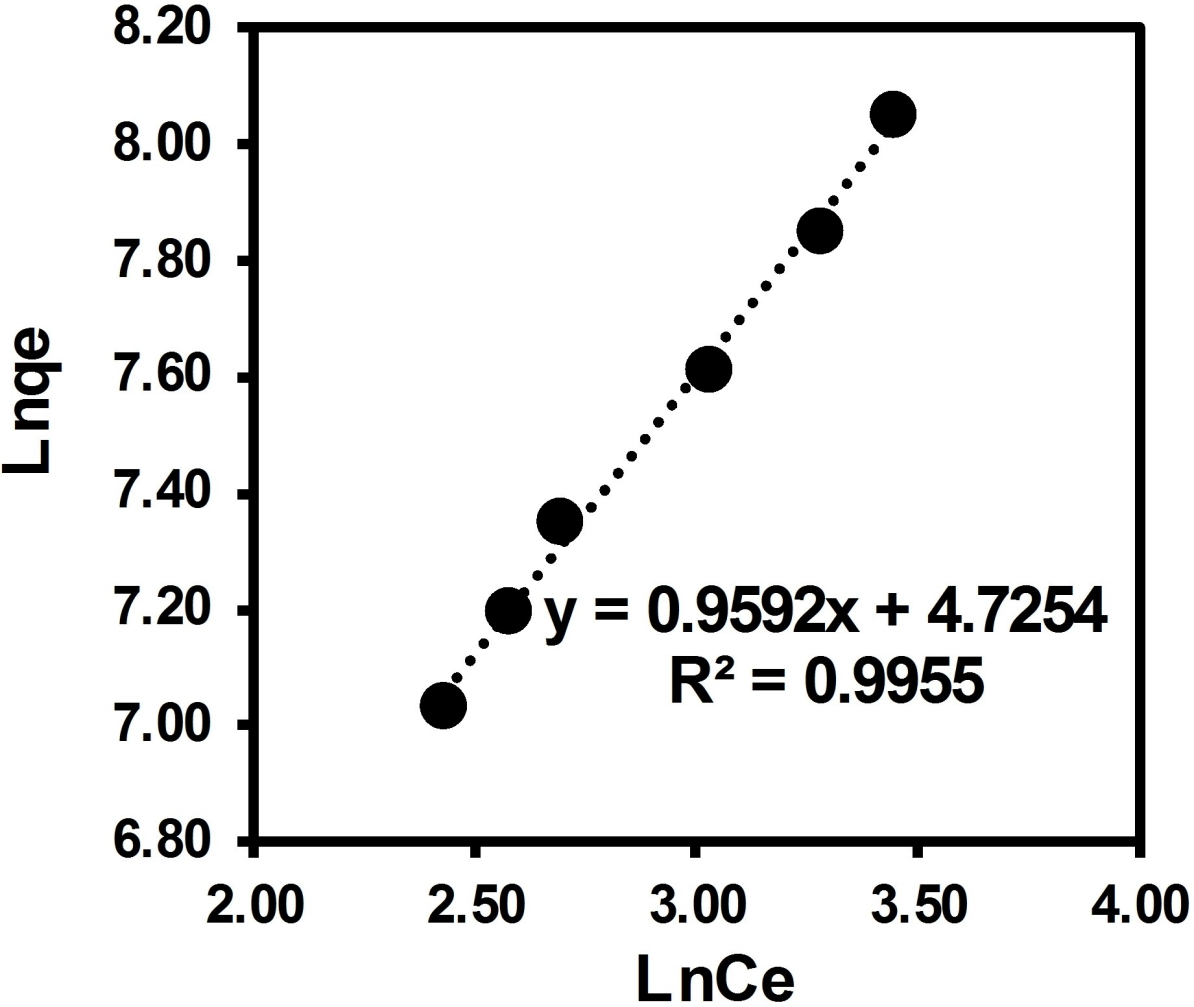
Freundlich isotherm for adsorption of ARS dye over BCFO.

In this study, the Langmuir, Temkin, and Freundlich isotherms were used to describe the adsorption of ARS solute onto surface of brownmillerite CaFeO_2.5_ in solution. The equilibrium data obtained with varying concentration of adsorbate over 10 mg of adsorbent was applied to the Langmuir, Temkin, and Freundlich model (Figures 13, 14, 15 with the adsorption parameters enclosed in Table 5). Langmuir separation factor “R_L_”, which is related to the affinity of binding sites, and thermodynamical parameter ΔG° were calculated using Eqs. 3 and 4, respectively (see the Materials and Methods section).

**Table 5:** Langmuir, Temkin, and Freundlich isotherm parameters, and thermodynamically equilibrium data for adsorption of ARS dye over BCFO in solution (SD represents the standard deviation).

Langmuir isotherm parameters							
	Slope	Intercept	R^2^	q_max_	K_L_	R_L_	ΔG° $\left(\frac{kJ}{mol}\right)$
	0.0396 (SD=0.0076)	1.5191 (SD=1.588)	0.8719	25.3	0.026	0.94	−9.04

Temkin isotherm parameters							
	Slope	Intercept	R^2^	K_T_ (L/mg)	B_T_ (kJ/mol)	–	–
	1958.3 (SD=123.39)	−3731.1 (SD=361.45)	0.9844	0.15	2	–	–

Freundlich isotherm parameters							
	Slope	intercept	R^2^	K_F_	n	–	–
	0.9592 (SD=0.0323)	4.7254 (SD=0.0946)	0.9955	113	1.043	–	–

According to the value of R_L_ in this current experiment (0.94), the nature of adsorption is predicted to be irreversible. In agreement with the electronic spectra studies, this can be related to the adsorbate‐adsorbent acid‐base interaction. Results are accounted for a favorable adsorption. The value of n=1.043 figures out that the adsorption is favorable with the Freundlich isotherm model.^[30]^ The Freundlich constant K_F_=113 is accounted for a suitable adsorption capacity of the adsorbent and shows a strong adsorption strength between adsorbent‐substrate. This can be related to the basic surface of the BCFO, showing suitable interaction with the acidic hydrogen of ARS dye. These results perfectly support above UV‐Vis and TPD studies.

The linear isothermal calculations and regression analysis predict that equilibrium data for the adsorption of ARS over Brownmillerite CaFeO_2.5_ in solution best fit with the Freundlich model (R^2^=0.9955) as compared to Temkin (R^2^=0.9844) and Langmuir models (R^2^=0.8719). It is also confirmed by the SD calculation, which is a measure of how spread out the data is from the average or mean value. The observed behavior implies that the degradation of ARS dye in solution over BCFO involves chemisorption through acid‐base interaction.

### Kinetic Studies

**Figure 16:**
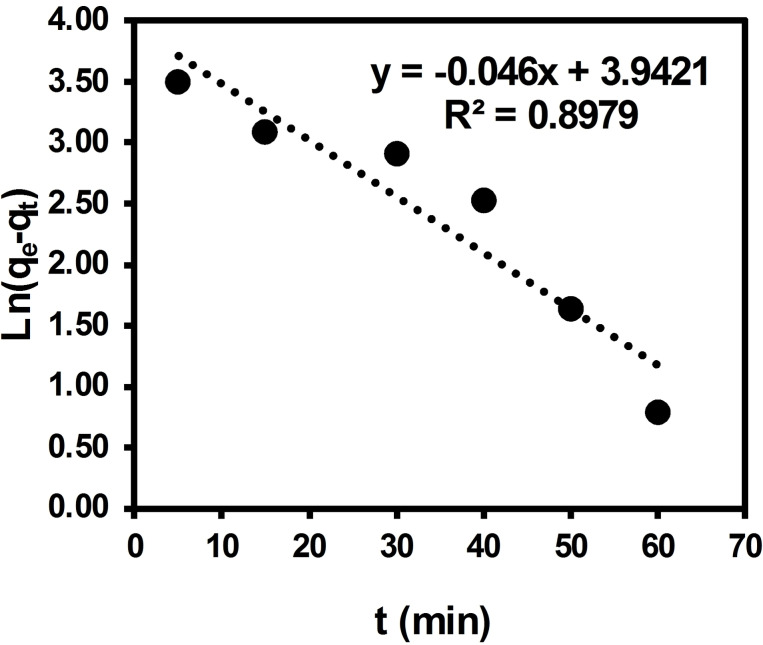
Test of pseudo‐first order equation for adsorption of ARS dye by BCFO.

Figure 16 displays a linear graph of Ln(q_e_−q_t_) plotted against ′t′ in accordance with the pseudo‐first order kinetic model of equilibrium data, as indicated by experimental findings.

**Figure 17:**
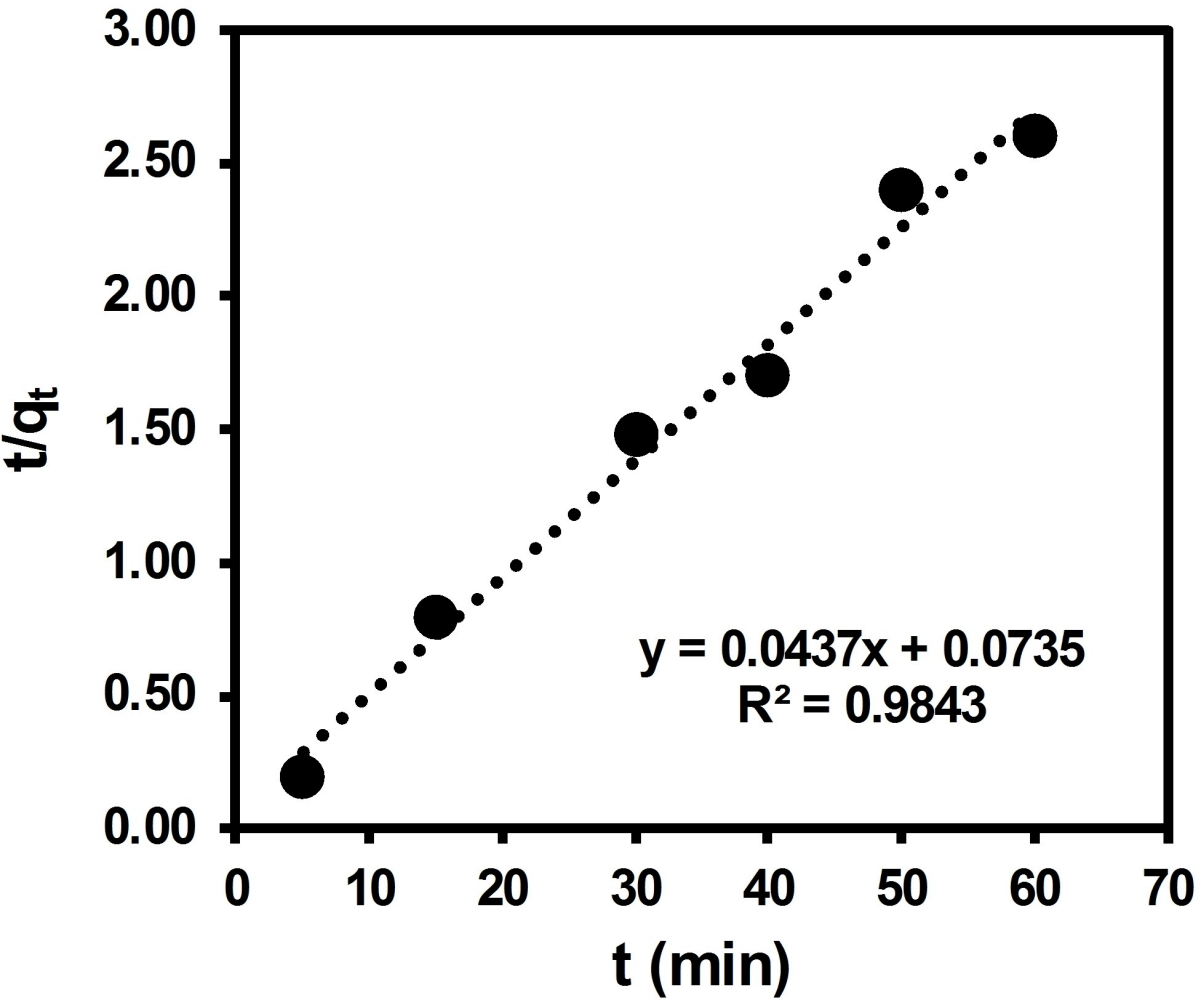
Test of pseudo‐second order equation for adsorption of ARS dye over BCFO.

Calculations have yielded standard deviations of 0.01 and 0.3 for the slope and intercept, respectively, in the pseudo‐first order kinetic plot. It is worth emphasizing that the Lagergren pseudo‐first order rate equation is a simplified model that may not provide a precise description of the adsorption kinetic of a solute onto a solid surface in all cases. As an alternative, the pseudo‐second‐order model was investigated, which may offer a more accurate fit to the experimental data. Thus, Figure 17 displays the plot of t/q_t_ versus 1/t, representing the pseudo‐second order kinetic model applied to equilibrium data. The standard deviations of the slope and intercept in the pseudo‐second order kinetic plot are 0.0028 and 0.11, respectively, as determined by calculations.

The correlation coefficient R^2^ value of 0.8979 indicates that the pseudo‐first order kinetic model is not suitable for describing the adsorption of ARS dye on BCFO. It means that the equilibrium of adsorption is influenced by both the quantity of dye and adsorbent concentrations. The suitability of the pseudo‐second order kinetic model is supported by the correlation coefficient R^2^ value, which is nearly equal to unity at 0.9843. The SD calculation, which measures the extent to which data deviates from the average or mean value, also provides confirmation. Results further confirm that both the concentration of dye and adsorbent quantities play a significant role in determining adsorption equilibrium. The equilibrium adsorption capacity, q_e_, and the pseudo‐second order rate constant (*k*_2_) were calculated to be 22.88 $\frac{mg}{g}$ and 2.6×10^−2^ $\frac{g}{mg\cdot min}$ , respectively.

## Conclusions

We have successfully synthesized brownmillerite type Ca_2_Fe_2_O_5_ nanoparticles, referred as BCFO by wet complexation. More importantly, its surface interacts suitably with alizarin Red S quinone dye through acid‐base interactions. This interaction is accompanied with a red shift of the visible peak of the ARS, which has been further confirmed by quantum chemical computations as a π→π^*^ transition from the HOMO to the LUMO. BCFO has been extensively characterized by X‐ray diffraction analysis, Fourier transform infrared spectroscopy, Compositional and Morphological Studies, CO_2_ temperature programmed desorption, analysis of the physico‐chemical properties, XPS spectroscopy, isothermal modeling of equilibrium data, as well as kinetic studies. Noticeably, CO_2_‐TPD analysis showed three different basic sites on the BCFO surface, one of which considered to be stronger than the others. With respect to ARS degradation, the catalyst degrades the ARS dye appropriately in the darkness barring any acidic, basic, or oxidant additives. FTIR and TOC analyses also support degradation. In addition, the porous structure of the prepared catalyst can be the cause of the proper degradation done over the catalyst. And this degradation is more accurately and precisely explained by the Freundlich isotherm and pseudo‐second‐order model. As a whole, results of this study have demonstrated that the BCFO is a promising material for the decomposition of ARS, or other persistent dyes that are difficult to degrade.

## Supporting Information Summary

The Supporting Information includes the Cartesian coordinates, energies and UV‐Vis data of computed ARS and ARS^‐1^; the energy dispersive spectrum of BCFO specimen; and extra data on the degradation of ARS solution.

## Conflict of interests

The authors declare no conflict of interest.

1

## Supporting information

As a service to our authors and readers, this journal provides supporting information supplied by the authors. Such materials are peer reviewed and may be re‐organized for online delivery, but are not copy‐edited or typeset. Technical support issues arising from supporting information (other than missing files) should be addressed to the authors.

Supporting Information

## Data Availability

The data that support the findings of this study are available in the supplementary material of this article.
