# Supplementary material for: Brownmillerite Calcium Ferrite, a Promising Perovskite‐Related Material in the Degradation of a Tight Dye under Ambient Conditions
Source: ChemistryOpen. 2023 Dec 5;13(3):e202300169. doi: 10.1002/open.202300169 (PMC10962484; doi:10.1002/open.202300169)
Supplement: Supplementary file 1 — Supporting Information [file OPEN-13-e202300169-s001.pdf]

ChemistryOpen

Supporting Information

Brownmillerite Calcium Ferrite, a Promising Perovskite-Related Material in the Degradation of a Tight Dye under Ambient Conditions

Zahra Noori, Azim Malekzadeh,* and Jordi Poater*

Contents

Table S1. Cartesian coordinates (in Å), total ADF energies (in kcal mol^{-1}), and UV-Vis data of the isomers of ARS.

Table S2. Cartesian coordinates (in Å), total ADF energies (in kcal mol^{-1}), and UV-Vis data of the isomers of ARS^{-1}.

Figure S1. Energy Dispersive Spectrum of BCFO specimen.

Figure S2. Degradation of the 100 ppm ARS solution in the presence of BCFO nanocatalyst.

Table S1. Cartesian coordinates (in Å), total ADF energies (in kcal mol^{-1}), and UV-Vis data of the isomers of ARS.

Alizarin Red S Isomers (ARS), ($C_{14}H_7O_7S$)		
**Right – Right H**	**Left – Right H**	**Left – left H**
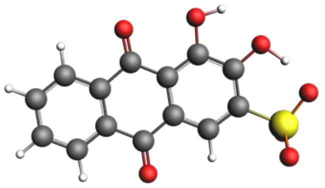	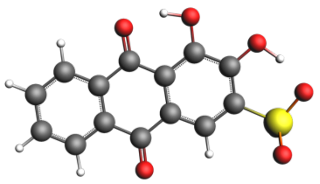	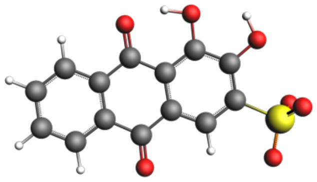
E = -5472.63 kcal/mol	E = -5479.06 kcal/mol	E = -5475.85 kcal/mol

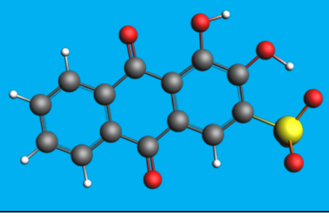

Symbol	x	y	z
S	2.87290783	-1.59066703	-3.13760615
O	1.18432812	2.83932193	-1.13282840
O	2.70321575	1.51297554	-2.77846384
O	-0.66230348	-2.61080108	0.64519960
O	-0.65744988	2.79174784	0.78334337
O	3.82958241	-0.60755856	-3.72241851
O	3.54083475	-2.63180082	-2.34695240
O	1.93760608	-2.12633237	-4.13800745
C	0.22144267	-0.59725724	-0.22944313
C	0.23649110	0.82336426	-0.20630533
C	-1.52238549	-0.63367615	1.61466676
C	-1.51197377	0.76861551	1.64227676
C	-0.65392025	-1.37844186	0.67270542
C	-0.63651400	1.56291396	0.73432851
C	1.87868849	-0.62831657	-1.98598791
C	1.03291735	-1.29689560	-1.10602011
C	1.09283010	1.48983319	-1.08654603
C	1.91756060	0.76124223	-1.97964692
C	-2.35794938	-1.33857024	2.48360674
C	-2.33968010	1.44420029	2.54117592
C	-3.17668381	-0.65826715	3.37353328
C	-3.16723851	0.73656009	3.40186219
H	0.99952919	-2.37699394	-1.09794399
H	-2.35469322	-2.41940035	2.45104705
H	-2.32468652	2.52514108	2.55514537
H	-3.82152312	-1.21030298	4.04557560
H	-3.80537942	1.26894443	4.09585902
H	1.84899895	3.05889322	-1.81001691
H	3.28085185	0.88425456	-3.29288968

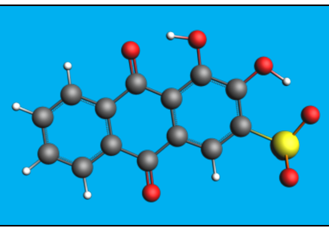

Symbol	x	y	z
S	4.46761195	-0.94439037	-0.02677418
O	1.18565895	3.00923857	0.31974408
O	3.63612499	2.05373877	0.29595605
O	-0.53538022	-2.73326760	-0.19304908
O	-1.32048258	2.60162699	0.23192170
O	5.39230871	0.14626726	0.39153596
O	4.47537650	-2.08260800	0.90059895
O	4.66017613	-1.33475349	-1.43196275
C	0.43120655	-0.58069197	-0.00407337
C	0.21752682	0.81595772	0.10841401
C	-2.07816407	-0.94076836	-0.07684190
C	-2.28179801	0.44609566	0.03340986
C	-0.70719557	-1.51669785	-0.09894613
C	-1.13530810	1.37193704	0.13140653
C	2.82034179	-0.21856742	0.06583741
C	1.72157015	-1.07572645	-0.02342491
C	1.31641165	1.67684317	0.20509450
C	2.63834603	1.15852893	0.18755393
C	-3.18025439	-1.79091136	-0.16664629
C	-3.58113555	0.95725818	0.05099184
C	-4.46934989	-1.27419304	-0.14836053
C	-4.67025088	0.10150821	-0.03964586
H	1.87391586	-2.14247182	-0.10728804
H	-3.01404077	-2.85605802	-0.25056387
H	-3.72561669	2.02536534	0.13567127
H	-5.31888464	-1.94164892	-0.21859796
H	-5.67522438	0.50371734	-0.02579351
H	0.20045042	3.17505757	0.31329430
H	4.48131941	1.53349686	0.34492400

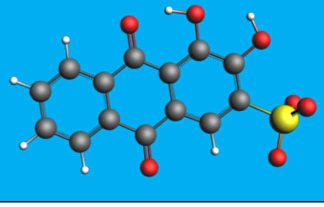

Symbol	x	y	z
S	4.49152049	−0.91607165	−0.03643272
O	1.20021648	3.01073117	0.08971466
O	3.66508101	2.01745073	0.05505448
O	−0.54403152	−2.74389662	−0.07309086
O	−1.31184929	2.60685604	0.08514885
O	5.13515077	−0.45417395	1.20726458
O	4.30450348	−2.37630943	−0.08070365
O	5.12905536	−0.37881276	−1.25262332
C	0.42793714	−0.58687539	−0.01404041
C	0.21052134	0.80924914	0.02736439
C	−2.08385707	−0.94533529	−0.01763386
C	−2.28767032	0.44589896	0.02303685
C	−0.71291060	−1.52455986	−0.03757335
C	−1.13937071	1.37254461	0.04819092
C	2.82273877	−0.22305389	−0.01074569
C	1.72274674	−1.08021948	−0.03257461
C	1.31183334	1.66817950	0.04941523
C	2.62666616	1.15370063	0.03117793
C	−3.18659708	−1.79834921	−0.04026466
C	−3.58652881	0.95755342	0.03979862
C	−4.47577798	−1.28112991	−0.02315262
C	−4.67636225	0.09805802	0.01673343
H	1.88519517	−2.14664994	−0.06454131
H	−3.02151803	−2.86647269	−0.07140943
H	−3.73040348	2.02865437	0.07087860
H	−5.32555043	−1.95164830	−0.04119125
H	−5.68130837	0.50018283	0.02965094
H	0.21672727	3.19305042	0.09852312
H	3.30571647	2.92119044	0.08282988

ARS isomers UV_Vis spectras

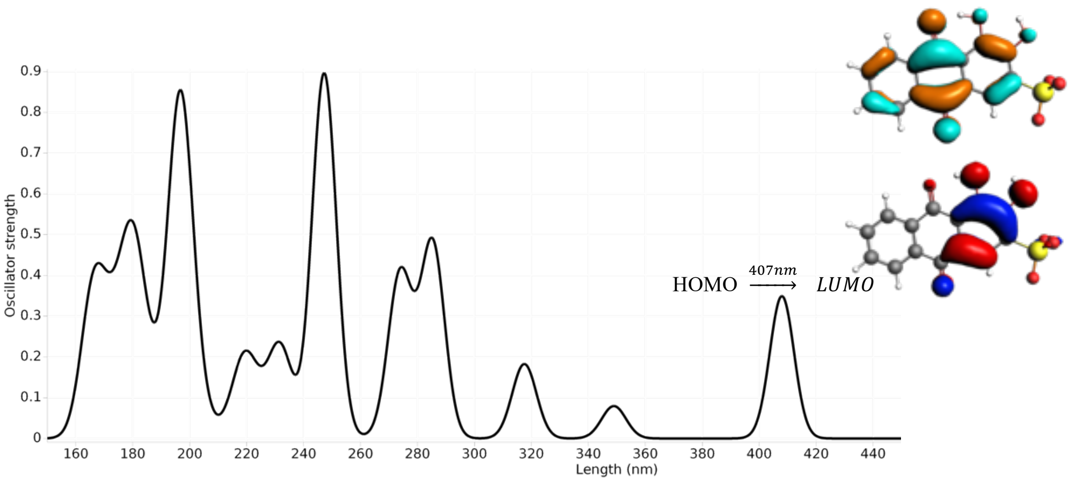

wavelength (nm) = 407	82a (HOMO) → 83a (LUMO)	$\Delta_{(\mathrm{HOMO-LUMO})\ (eV)} = 5.30$

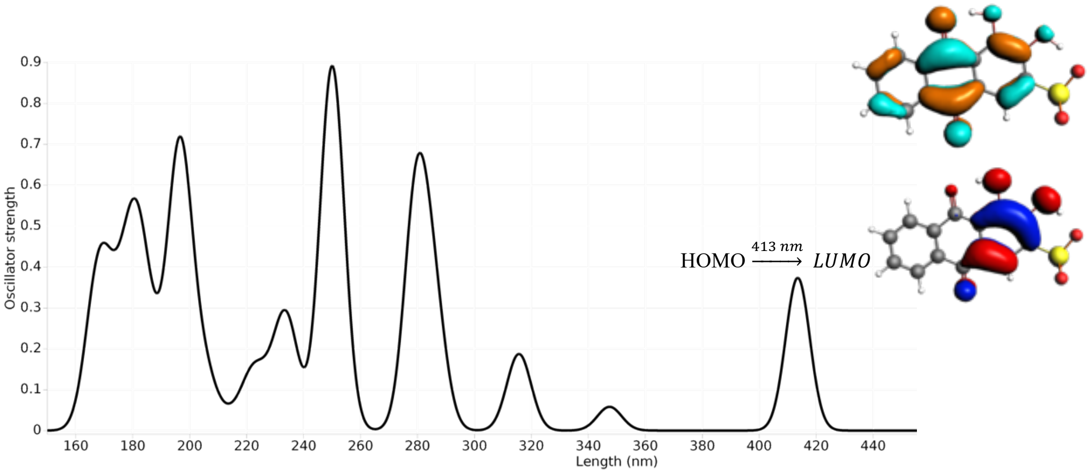

wavelength (nm) = 413	82a (HOMO) → 83a (LUMO)	$\Delta_{(\mathrm{HOMO-LUMO})\ (eV)} = 5.25$

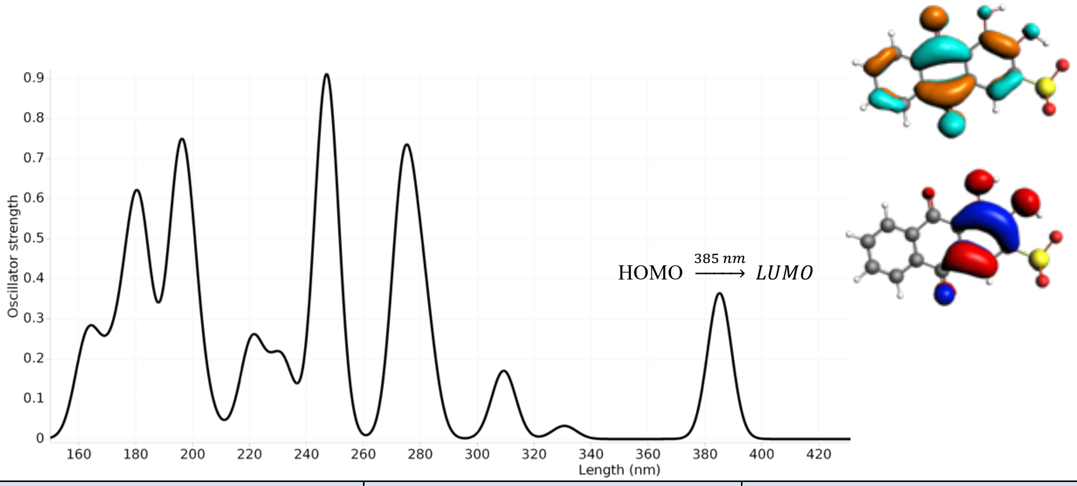

wavelength (nm) = 385	82a (HOMO) → 83a (LUMO)	$\Delta_{(\mathrm{HOMO-LUMO})\ (eV)} = 5.49$

Table S1. Cartesian coordinates (in Å), total ADF energies (in kcal mol^{-1}), and UV-Vis data of the isomers of ARS^{-1}.

ARS^- (mono-protic), ($C_{14}H_6O_7S$)	
HL	HR
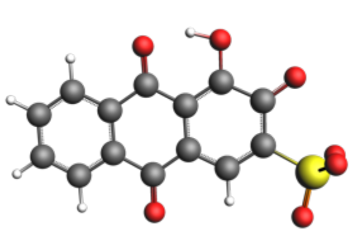	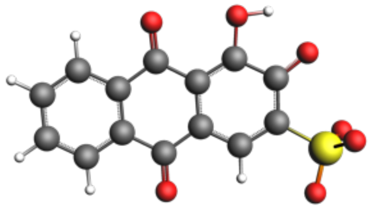
E = -5467.91 kcal/mol	E = -5465.69 kcal/mol
2HL	2HR
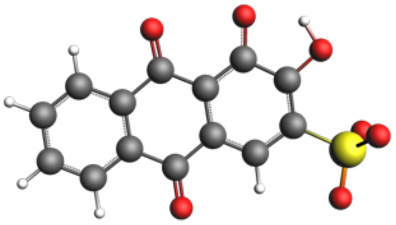	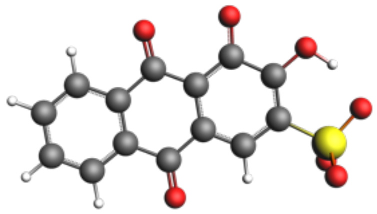
E = -5463.92 kcal/mol	E = -5461.73 kcal/mol

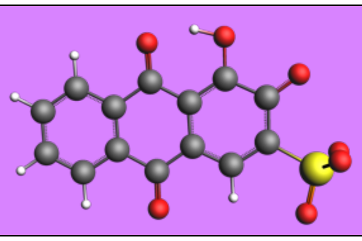

Symbol	x	y	z
S	3.00269152	−1.41424696	−3.13407879
O	0.91536416	2.87248994	−1.17007209
O	2.57662501	1.61670508	−2.84570415
O	−0.53540815	−2.68593838	0.64818399
O	−0.82244281	2.74100773	0.66233198
O	4.38059526	−0.97261718	−2.82176543
O	2.80165171	−2.85140457	−2.84283603
O	2.59735160	−1.06568122	−4.51447649
C	0.23013398	−0.63691982	−0.26472694
C	0.15372132	0.79300568	−0.25955434
C	−1.50082100	−0.71949717	1.55388166
C	−1.58062065	0.68481555	1.56345686
C	−0.57920622	−1.43983234	0.62640015
C	−0.74703111	1.48927156	0.64850796
C	1.90513237	−0.53103823	−2.02697130
C	1.10860502	−1.25305491	−1.15365284
C	0.94978410	1.52834903	−1.13343664
C	1.87890892	0.89936269	−2.07564330
C	−2.30203996	−1.45239217	2.43102097
C	−2.45566405	1.32648657	2.44491808
C	−3.16860076	−0.80735245	3.30335126
C	−3.24648524	0.58585284	3.31101168
H	1.16771238	−2.33177644	−1.15716584
H	−2.23454297	−2.53155919	2.41748677
H	−2.50618673	2.40667688	2.44062410
H	−3.78503765	−1.38764057	3.97872605
H	−3.92238034	1.08875393	3.99113169
H	0.24173645	3.13344987	−0.47673525

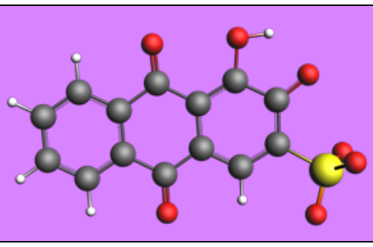

Symbol	x	y	z
S	3.53291787	−0.44635928	−2.81410743
O	1.13813499	3.10082763	−0.02614175
O	2.96720464	2.38794339	−1.62391398
O	−0.55015089	−2.69491221	−0.28422935
O	−1.01668572	2.42398636	1.46064941
O	4.82565215	−0.16814950	−2.14962571
O	3.31409054	−1.89333836	−3.02810360
O	3.36007872	0.33557039	−4.05855034
C	0.29638726	−0.48179270	−0.34087143
C	0.18387345	0.87742803	0.11040959
C	−1.72149395	−1.11260089	1.02853553
C	−1.84123689	0.21159003	1.47842863
C	−0.63268175	−1.51184587	0.09396604
C	−0.88734424	1.26993523	1.04052801
C	2.24762780	0.08416341	−1.68695475
C	1.31789203	−0.83360984	−1.21956806
C	1.11195198	1.79288733	−0.35666571
C	2.18689537	1.43920463	−1.27909740
C	−2.63814167	−2.07219694	1.46488115
C	−2.87404990	0.55132280	2.35592486
C	−3.66117390	−1.72532077	2.33621491
C	−3.77987480	−0.40884239	2.78348326
H	1.38335703	−1.86132417	−1.54541319
H	−2.53482393	−3.08882406	1.11096841
H	−2.95419122	1.57507236	2.69501561
H	−4.36666934	−2.47686057	2.66841665
H	−4.57732388	−0.13580037	3.46339065
H	1.92424899	3.41607445	−0.54605222

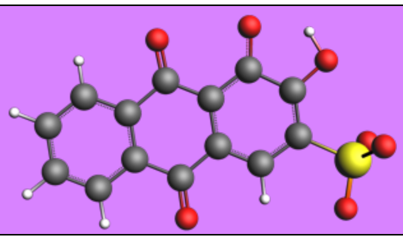

Symbol	x	y	z
S	4.48443142	−0.95805184	−0.04552909
O	1.36021485	3.03140836	0.09762187
O	3.66388998	2.02616754	0.05365314
O	−0.51862024	−2.71238403	−0.05646352
O	−1.39771750	2.63777736	0.09078392
O	5.15708034	−0.51551441	1.19373347
O	4.27572721	−2.41845070	−0.08965120
O	5.13987227	−0.43874662	−1.26394540
C	0.42679982	−0.53266474	−0.00611709
C	0.20836811	0.88510571	0.03583004
C	−2.07177970	−0.93306547	−0.01348868
C	−2.28353262	0.45300531	0.02416055
C	−0.69594836	−1.48659924	−0.02762044
C	−1.14310687	1.42270109	0.05442104
C	2.83761332	−0.23223643	−0.01129895
C	1.70423167	−1.06016476	−0.02843787
C	1.33856680	1.75175577	0.05745359
C	2.66050348	1.13074322	0.03142990
C	−3.16597562	−1.80315658	−0.03908968
C	−3.59150661	0.94370269	0.03431593
C	−4.46022679	−1.30504123	−0.02864054
C	−4.67284402	0.07409354	0.00780720
H	1.83119568	−2.13111403	−0.06001703
H	−2.98427122	−2.86894501	−0.06763006
H	−3.74472219	2.01368792	0.06319114
H	−5.30315327	−1.98462197	−0.04928028
H	−5.68218174	0.46691772	0.01569681
H	3.15020955	2.88072752	0.08282066

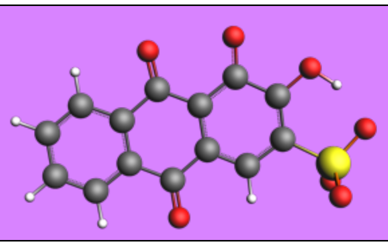

Symbol	x	y	z
S	4.43560702	−0.94765822	0.01553374
O	1.23960648	3.06460713	0.30731778
O	3.66201774	2.07766750	0.27463582
O	−0.48434826	−2.69978315	−0.18815664
O	−1.46351819	2.62108370	0.21485694
O	5.41825170	0.16068470	0.19387179
O	4.47745406	−1.91538051	1.12515711
O	4.55227484	−1.58829437	−1.30631094
C	0.41918464	−0.50865418	0.00119884
C	0.18695919	0.90368214	0.11119859
C	−2.06887030	−0.95212950	−0.07524307
C	−2.29729324	0.42714889	0.03045519
C	−0.68657963	−1.48102231	−0.09441222
C	−1.17155117	1.41452025	0.12648277
C	2.81690498	−0.16917625	0.06874747
C	1.69888802	−1.01660303	−0.01914207
C	1.30568132	1.80482283	0.20550644
C	2.64607058	1.19631715	0.18089390
C	−3.15005026	−1.83528173	−0.16221445
C	−3.61292278	0.89801900	0.04700379
C	−4.45080031	−1.35611564	−0.14469916
C	−4.68156689	0.01686117	−0.03948905
H	1.83575948	−2.08516502	−0.10365301
H	−2.95211905	−2.89555086	−0.24291056
H	−3.78151026	1.96291831	0.12837567
H	−5.28426780	−2.04427294	−0.21231077
H	−5.69611845	0.39608115	−0.02540354
H	4.50040104	1.54782559	0.26042782

ARS$^-$ UV_Vis spectras

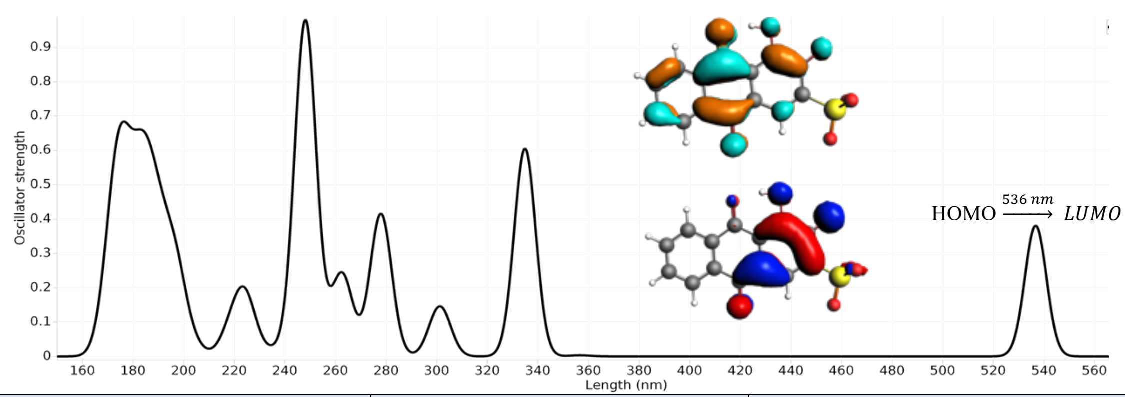

wavelength (nm) = 536	82a (HOMO) → 83a (LUMO)	$\Delta_{(HOMO-LUMO)\ (eV)} = 4.46$

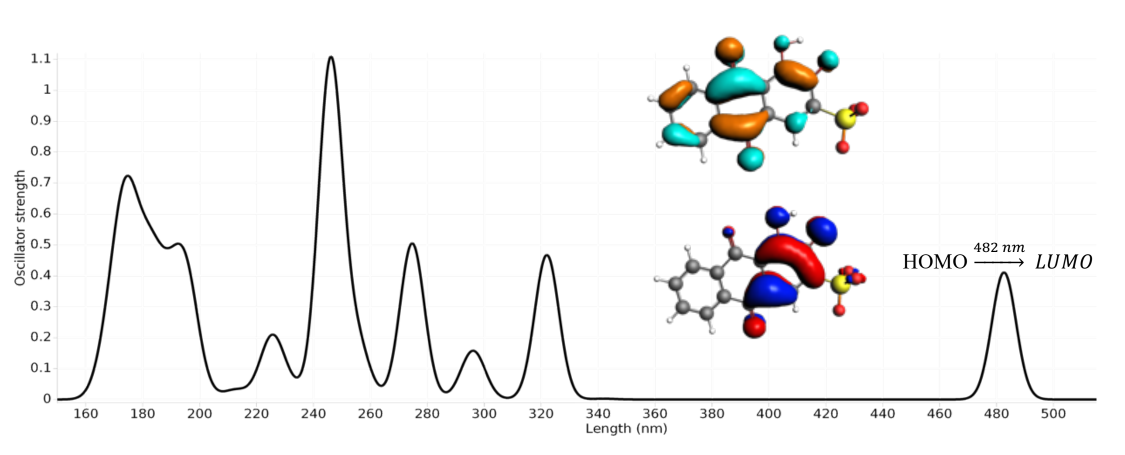

wavelength (nm) = 482	82a (HOMO) → 83a (LUMO)	$\Delta_{(HOMO-LUMO)\ (eV)} = 4.73$

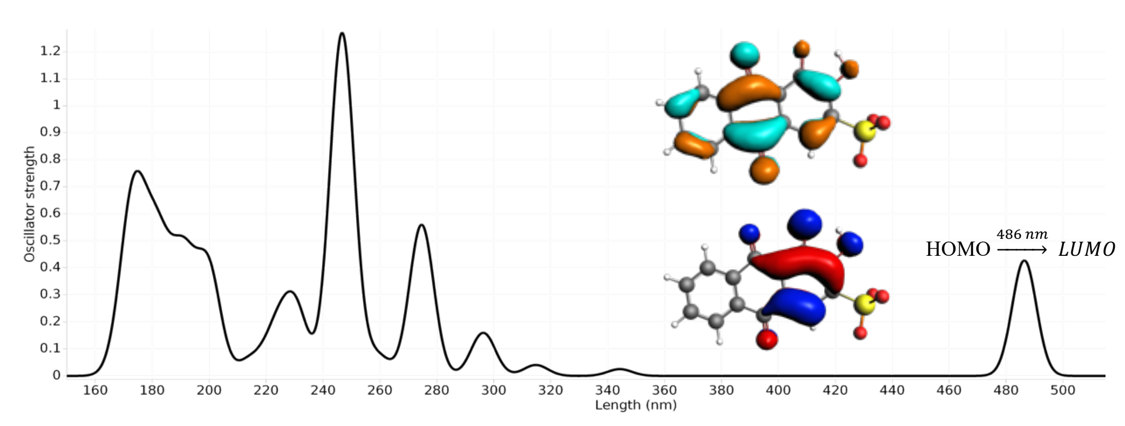

wavelength (nm) = 486	82a (HOMO) → 83a (LUMO)	$\Delta_{(HOMO-LUMO)\ (eV)} = 4.74$

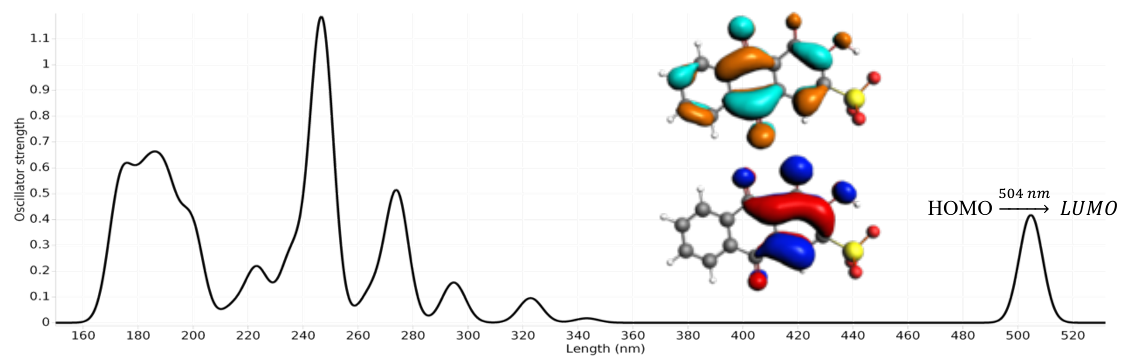

wavelength (nm) = 504	82a (HOMO) → 83a (LUMO)	$\Delta_{(HOMO-LUMO)\ (eV)} = 4.66$

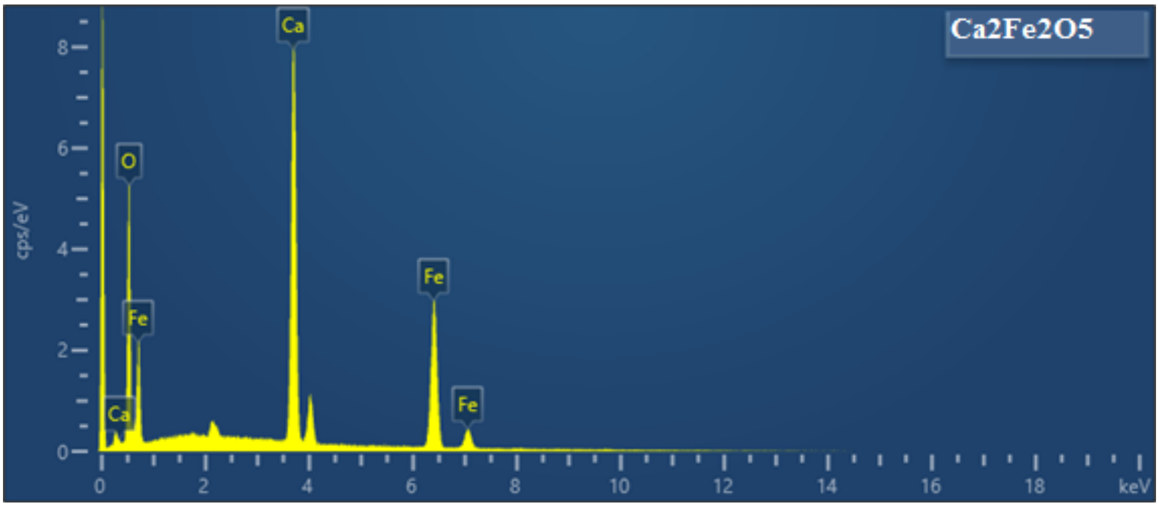

Figure S1. Energy Dispersive Spectrum of BCFO specimen.

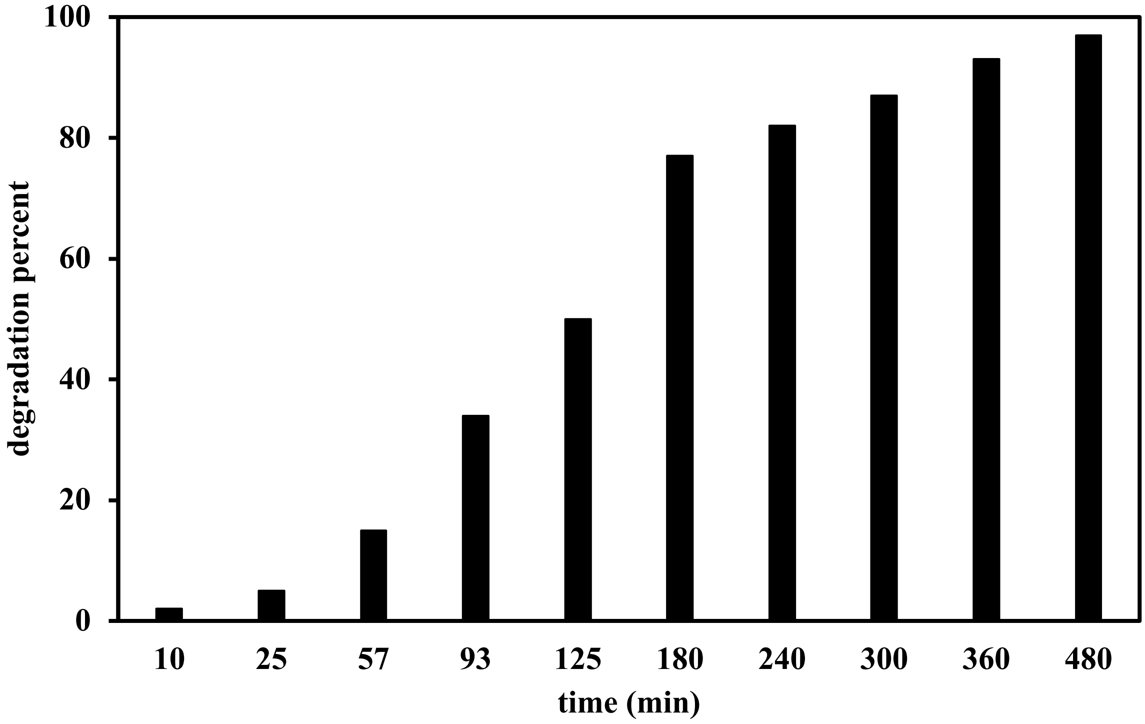

Figure S2. Degradation of the 100 ppm ARS solution in the presence of BCFO nanocatalyst.
